# Quantum Defects as a Toolbox for the Covalent Functionalization of Carbon Nanotubes with Peptides and Proteins

**DOI:** 10.1002/anie.202003825

**Published:** 2020-07-13

**Authors:** Florian A. Mann, Niklas Herrmann, Felipe Opazo, Sebastian Kruss

**Affiliations:** ^1^ Institute of Physical Chemistry Georg-August Universität Tammannstraße 6 37077 Göttingen Germany; ^2^ Center for Biostructural Imaging of Neurodegeneration Von-Siebold-Straße 3a 37075 Göttingen Germany

**Keywords:** bioconjugation, nanobodies, nanotubes, peptides, quantum defects

## Abstract

Single‐walled carbon nanotubes (SWCNTs) are a 1D nanomaterial that shows fluorescence in the near‐infrared (NIR, >800 nm). In the past, covalent chemistry was less explored to functionalize SWCNTs as it impairs NIR emission. However, certain sp^3^ defects (quantum defects) in the carbon lattice have emerged that preserve NIR fluorescence and even introduce a new, red‐shifted emission peak. Here, we report on quantum defects, introduced using light‐driven diazonium chemistry, that serve as anchor points for peptides and proteins. We show that maleimide anchors allow conjugation of cysteine‐containing proteins such as a GFP‐binding nanobody. In addition, an Fmoc‐protected phenylalanine defect serves as a starting point for conjugation of visible fluorophores to create multicolor SWCNTs and in situ peptide synthesis directly on the nanotube. Therefore, these quantum defects are a versatile platform to tailor both the nanotube's photophysical properties as well as their surface chemistry.

## Graphical abstract

**Two new quantum defects** were incorporated into single‐walled carbon nanotubes (SWCNT) carrying anchor groups for functionalization with biomolecules. The potential and versatility of this approach was demonstrated by the conjugation of a GFP‐binding nanobody as well as the growth of (fluorescent) peptide chains directly on the nanotube's carbon lattice.

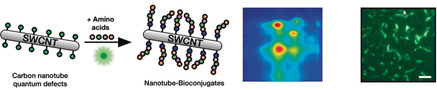

## Introduction

Since their discovery^1^ single‐walled carbon nanotubes (SWCNTs) attracted a lot of attention not only because of their unique chemical structure, but also because of their outstanding photophysical properties such as non‐bleaching/blinking near‐infrared (NIR) fluorescence.^2^, ^3^, ^4^ This NIR fluorescence is beneficial especially for bioimaging as the emission wavelength of SWCNTs falls into the so‐called tissue‐transparency window where the absorption of water, hemoglobin, and lipids reaches a combined minimum and scattering is reduced compared to that of visible light.^5^ Consequently, SWCNTs already found application in diverse settings ranging from in vivo NIR imaging^6^, ^7^, ^8^ over drug delivery vehicles^9^, ^10^ to NIR optical sensors.^11^, ^12^, ^13^, ^14^, ^15^, ^16^, ^17^, ^18^, ^19^, ^20^ In order to carry out their desired function in these important applications, the pure carbon tube has to be modified with, for example, the cargo to be carried or a recognition unit imparting specificity for the analyte to be detected. Furthermore, carbon nanotubes are not water‐soluble, preventing applications in aqueous systems unless the hydrophobic surface is coated with an amphiphilic surfactant.^12^ In the last 20 years, both covalent and noncovalent functionalization have been employed for the decoration of SWCNTs with functional units. Noncovalent functionalization such as adsorption of DNA is by far the most frequently applied approach owing to its ease‐of‐use and mild conditions.^21^, ^22^, ^23^ Furthermore, conformational changes of the coating molecule can directly translate into changes of the NIR fluorescence, which is interesting for sensing.^16^, ^24^ On the other hand, covalent functionalization leads to more stable conjugates, but destroys the SWCNT's extended π‐network and thus also the NIR fluorescence.^2^, ^25^ In 2017, Setaro et al. reported preserved fluorescence by using a [2+1]‐cycloaddition with electron‐poor aromatic nitrenes, which they also used for the attachment of gold nanoparticles and spiropyranes.^26^ Very recently, Godin et al. used spyropyran‐switchable SWCNTs for NIR superresolution microscopy,^27^ while Chio et al. used the same nitrene [2+1]‐cycloaddition for the attachment of small molecules such as biotin.^28^

However, sp^3^ defects can not only diminish the NIR fluorescence, but were also found to modulate it depending on the nature and density of the defects. In 2010, Ghosh et al. reported on a NIR emission peak red‐shifted by approximately 130 nm (termed E_11_^*^) upon introduction of oxygen defects.^29^ Later, Piao et al. observed a similar peak shift and enhanced fluorescence quantum yield upon introduction of aryl defects using diazonium salts (quantum defects).^30^ This technique also enabled tuning of the defect fluorescence both in terms of intensity and emission wavelength via different substituents on the aryl/alkyl defect.^31^, ^32^, ^33^, ^34^

In the last years, a number of different oxygen and aryl defects were reported that are now very promising tools for the generation of brighter/modified SWCNTs,^31^, ^35^, ^36^ pH^37^ and saccharide^38^ sensors, short fluorescent SWCNTs for superresolution microscopy,^39^ and single‐photon sources for quantum computing.^40^, ^41^ Furthermore, this new defect‐induced fluorescence feature moves the emission even further into the biotransparency window, leading to even better tissue penetration properties.^42^, ^43^

## Results and Discussion

**Figure 1:**
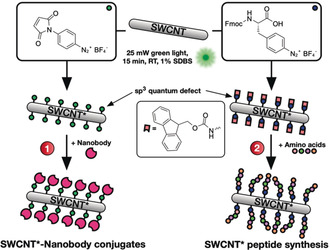
Defects as a generic handle to functionalize SWCNTs. Differently substituted aryl defects are introduced into the nanotube's sidewall and used to conjugate biomolecules such as nanobodies (1) or for the direct growth of peptide chains on the SWCNT* (2). Fmoc=fluorenylmethoxycarbonyl.

In this work, we expand the use of quantum defects from modulation of the SWCNTs’ photophysical properties towards using them as modular anchors for the attachment of peptides and proteins (Figure 1). To this end, we employed an (*N*‐maleimido)phenyldiazonium salt (MalPh‐Dz, **2**), which—after defect introduction—can be targeted by thiol‐containing molecules such as proteins, while at the same time generating a further red‐shifted E_11_^*^ emission feature (defect‐carrying SWCNTs are thus referred to as SWCNT* in the following).

**Figure 2:**
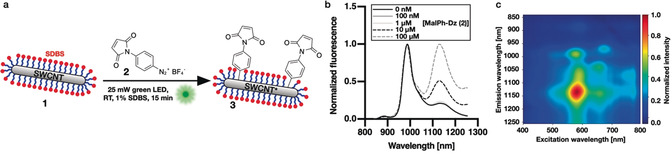
Light‐driven introduction of maleimide‐carrying quantum defects into SWCNTs. a) Schematic of the photoinduced introduction of defects based on aqueous diazonium chemistry. Size of the structures is not to scale. b) NIR ‐fluorescence spectra of SWCNT*/SDBS samples after the photoinduced defect reaction (15 min illumination) with different concentrations of MalPh‐Dz. c) NIR fluorescence excitation–emission map after introduction of the covalent MalPh defects, removal of excess **2**, and resuspension in 1 % SDBS (a control 2D spectrum can be found in Figure S3).

We started our investigations by optimizing the narrow window of reaction conditions^44^ between sodium dodecylbenzenesulfonate (SDBS)‐dispersed SWCNTs **1** and 4‐(*N*‐maleimido)phenyldiazonium tetrafluoroborate (MalPh‐Dz, **2**), allowing for the NIR fluorescence to be altered but not diminished (Figure 2 a). For a fast and efficient screening of conditions (reaction time and reactant concentration, see Figure S1), we made use of a 96‐well green LED array for SWCNT excitation driving the radical arylation reaction. The best results were achieved when a 10 nm SWCNT (c_carbon_≈530 μm) solution (dispersed in 1 % SDBS/H_2_O) was mixed with **2** (100 μm) and irradiated for 15 minutes. Higher MalPh‐Dz concentrations would lead to too many defects and diminish NIR fluorescence.^30^

The success of the defect reaction was monitored by NIR fluorescence spectroscopy (Figure 2 b) and the SWCNTs’ structural integrity and colloidal stability was controlled using Vis/NIR absorbance spectroscopy (Figure S2). The fluorescence spectra and the excitation/emission map (see Figure 2 c) clearly show a growing E_11_^*^ peak at approximately 1135 nm with increasing concentration of **2** (control 2D spectrum with a nonmodified SWCNT/SDBS sample can be found in Figure S3). The increased E_11_^*^/E_11_ ratio of the 2D versus the 1D NIR fluorescence spectrum could be attributed to prolonged exposure to MalPh‐Dz **2** before spin filtration and measurement of the 2D spectrum or due to preferential redispersion of MalPh‐SWCNT*.

After successful introduction of (*N*‐maleimido)phenyl quantum defects (MalPh defects), we tested whether biomolecules can be conjugated to this anchor. As a first example, we chose a nanobody against green fluorescent protein (GFP). Nanobodies are the isolated antigen‐binding region of heavy‐chain antibodies found, for example, in *Camelidae* and are only 10 % of the size of conventional antibodies.^45^, ^46^ This renders them very useful as binders for diverse applications such as superresolution microscopy,^47^, ^48^, ^49^ live‐cell immunostaining after modification with cell‐penetrating peptides,^50^ and isotopic labeling of biological samples for secondary‐ion mass spectrometry (SIMS) imaging.^51^ Nanobodies binding GFP (GFP‐binding protein, GBP) in particular can be used as a platform technology due to the widespread availability of GFP‐fusion proteins or even whole genetically modified organisms expressing GFP‐fusion proteins, giving the possibility to target a whole variety of proteins with just one single conjugate. Similar to our previous (noncovalent) work,^13^ we used a GBP with a single ectopic C‐terminal cysteine for oriented conjugation to the MalPh‐SWCNT* **3** leaving the antigen‐binding region pointing away from the SWCNT* surface.

**Figure 3:**
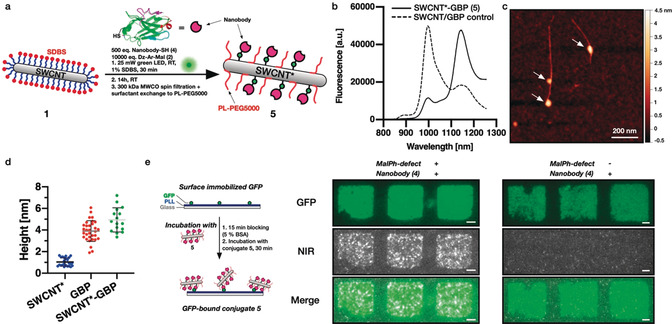
One‐pot introduction of quantum defects and nanobody conjugation to SWCNT*. a) Schematic of the one‐pot defect reaction and bioconjugation to GFP‐binding nanobodies (GBP). b) NIR fluorescence spectra comparing the SWCNT*‐GBP conjugate **5** and its negative control where no MalPh‐Dz was added, showing the successful introduction of quantum defects despite the simultaneous addition of the anti‐GFP nanobody. c) Atomic force microscopy (AFM) image of a SWCNT after defect/conjugation reaction to GBP. Scale bar=200 nm. The arrows point at locations of increased height indicating the conjugation of the nanobody. The reaction conditions (low defect density, reactant concentration) were chosen to immobilize only a few GBPs per tube. d) Measured heights of SWCNTs* only noncovalently wrapped by phospholipid‐polyethyleneglycol‐5000 (PL‐PEG5000), isolated GBP nanobodies, and SWCNT*‐GBP show the increased height after nanobody conjugation (mean ± SD, *n* ≥16). e) Pattern of green fluorescent protein (GFP) on a glass surface incubated with SWCNT*‐GBP. The colocalization shows retained functionality of the nanobody even after covalent conjugation to the SWCNT. Scale bars=5 μm.

With the fast hydrolysis kinetics of *N*‐aryl maleimides^52^ in mind, we evaluated both the sequential defect introduction followed by nanobody conjugation as well as a one‐step approach combining all three reaction partners at once (Figure 3 a). For the sequential reaction, the excess diazonium salt **2** was removed using 300 kDa molecular weight cutoff (MWCO) spin filters followed by resuspension of the now naked MalPh‐SWCNT* **3** in 1x phosphate‐buffered saline (PBS, pH 7.4) and reaction with 500 equiv. (≈25 equiv. with respect to introduced maleimides) of the nanobody **4** (16 h at room temperature). In the one‐step approach, the same excess of GBP was added directly during the defect introduction (30 minutes instead of 15 minutes for the sequential reaction) and left to react for 16 h at room temperature as well. After defect‐introduction/bioconjugation, the excess nanobody was removed using 300 kDa‐MWCO‐spin‐filtration and the SWCNT*‐GBP conjugate **5** resuspended in 1xPBS using 1,2‐distearoyl‐*sn*‐glycero‐3‐phosphoethanolamine‐*N*‐[methoxy(polyethylene glycol)‐5000] (ammonium salt, PL‐PEG5000). As shown in Figure S4, the redispersion step was efficient only for the conjugate **5** synthesized via the one‐step approach, yielding a highly concentrated solution (OD=1.8). Consequently, we proceeded with the resuspended conjugate **5** resulting from the one‐step procedure in the following.

Figure 3 b shows the NIR fluorescence spectra of the conjugate **5** as well as the negative control, where no diazonium salt **2** was added, both resuspended using PL‐PEG5000. A comparison of both spectra shows successful introduction of sp^3^ quantum defects and E_11_^*^ emission. To further evaluate whether these defects also contain the covalently attached nanobody, we employed atomic force microscopy (AFM). Here, the one‐step‐synthesized sample clearly shows nanobodies attached to the SWCNT* (see Figure 3 c, control without **2** in Figure S5) with the additional height introduced by the GBP fitting both the value measured by AFM (*d*=4.3±0.9 nm) as well as the diameter obtained from the crystal structure (PDB: 3G9A, *d*=3.4–4.3 nm, see Figure S6). In a few cases, also larger heights of SWCNT*‐GBP conjugates (approximately 7 nm) were measured, which could be explained by the possible side reaction of the diazonium salt **2** with the GBP′s aromatic residues leading to dimer formation. Taken together, these results indicate successful conjugation of the nanobody to maleimide‐bearing quantum defects, which in turn are still able to modulate the SWCNT*’s NIR fluorescence, yielding emission at 1143 nm.

As a next step, we verified that the nanobody is still able to bind GFP even after covalent conjugation to the SWCNT*. Therefore, we immobilized GFP (patterned using polydimethylsiloxane (PDMS)‐based microcontact printing) on a poly‐l‐lysine (PLL)‐coated glass surface, followed by blocking (with bovine serum albumin [BSA])/washing steps and incubation with the conjugate **5**.

The observed colocalization of the GFP and the NIR channel indicates retained function of the GBP even after covalent conjugation to the SWCNT* (Figure 3 e, control without the MalPh defect on the right and without GBP in Figure S7). This is, to the best of our knowledge, the first covalent conjugation of a functional (immuno)protein to a SWCNT under preserved/enhanced NIR fluorescence.

After having successfully established MalPh quantum defects as an anchor for the attachment of (immuno)proteins, we wanted to challenge this defect‐based approach even further with the aim of synthesizing peptide chains directly on the SWCNT sidewall. While there are a few reports on the use of coiled‐coil or cyclic peptides for SWCNT dispersion^53^, ^54^, ^55^ and the noncovalent immobilization of RGD motifs,^56^ covalent immobilization of peptides is less explored. Pantarotto et al. and Bianco et al. utilized the 1,3‐dipolar cycloaddition of azomethine ylides for the covalent modification of SWCNTs and for subsequent attachment of previously synthesized, short peptides.^57^, ^58^ However, this approach destroys the SWCNT's optical properties and rules out NIR fluorescence imaging applications.

**Figure 4:**
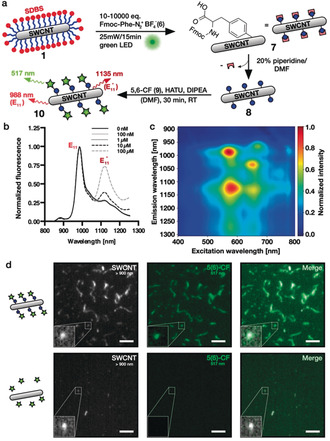
Fmoc‐phenylalanine quantum defects and multicolor SWCNTs. a) Strategy for defect introduction and subsequent Fmoc deprotection followed by CF conjugation. DIPEA=*N,N*‐diisopropylethylamine, HATU=1‐[bis(dimethylamino)methylene]‐1*H*‐1,2,3‐triazolo[4,5‐b]pyridinium 3‐oxide hexafluorophosphate. b) NIR fluorescence spectra of SWCNTs treated with different concentrations of Fmoc‐Phe‐Dz showing increased E_11_*/E_11_ ratios at higher diazonium salt concentrations. c) Excitation–emission map of Fmoc‐Phe‐SWCNT* showing the E_11_* fluorescence amongst other minor SWCNT species and E_11_ fluorescence. d) SWCNT*‐Phe‐CF immobilized on a glass slide showing colocalization of the NIR (>900 nm) and the CF channels (500–550 nm), whereas the control without sp^3^ defects does not show a CF signal, indicating successful conjugation. Scale bars=5 μm.

To use quantum defects as a starting point for peptide growth, we synthesized a diazonium salt containing a fluorenylmethoxycarbonyl (Fmoc)‐protected l‐phenylalanine (Fmoc‐Phe‐Dz, **6**) in a one‐step procedure. Next, we again optimized the conditions for defect introduction (Fmoc‐Phe defects) and evaluated the success using 1D/2D NIR fluorescence spectroscopy (Figure 4 b,c, control 2D spectrum in Figure S8). We carried out the Fmoc deprotection using 20 % piperidine/DMF in a 1 mL syringe reactor equipped with a standard 20 μm pore‐size frit. After washing, we coupled the fluorophore 5(6)‐carboxyfluorescein (CF) to assess addressability of the unprotected amine. Figure 4 d shows colocalization of the NIR and the CF channels after immobilization on glass and washing steps using 1xPBS with 0.1 % Triton‐X‐100 as opposed to the negative controls [without **6** (Figure 4 d) or without Fmoc deprotection (Figure S9)]. This result shows that the unprotected amine is still addressable and the conjugation of carboxyfluorescein led to the generation of covalently linked multicolor SWCNTs*.

**Figure 5:**
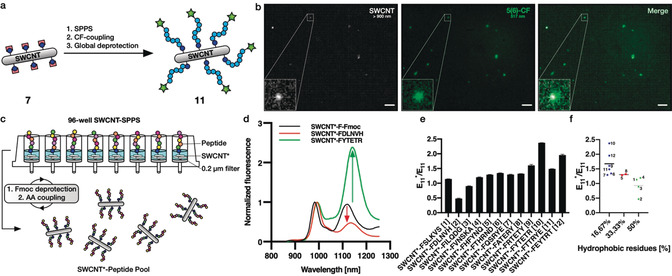
In situ peptide synthesis and modulation of E_11_* peak intensities. a) Strategy for the generation of covalent and fluorescent SWCNT*‐F‐R_6_‐CF conjugates based on Fmoc/O^t^Bu‐solid‐phase peptide synthesis (SPPS) followed by N‐terminal CF coupling and Pbf deprotection of the arginine side chains. b) SWCNT*‐R_6_‐CF spin‐coated on a glass coverslip showing colocalization of the NIR and the CF channels, indicating successful peptide synthesis and N‐terminal CF coupling directly on the SWCNT sidewall (scale bars=10 μm). c) 96‐Well peptide synthesis for the generation of a SWCNT‐peptide pool following the same Fmoc/O^t^Bu‐SPPS protocol as shown in (a), yet here in a 96‐well plate with filters (0.2 μm pore size). d) Normalized NIR‐PL spectra before and after synthesis of two selected peptide sequences showing the modulation of the defect‐induced fluorescence. The red‐shift from the protected to the unprotected sample could be attributed to the different surfactants (SWCNT*‐F‐Fmoc: SDBS, SWCNT*‐Peptide: DOC). e) The SWCNT fluorescence properties (in particular the E_11_*/E_11_ ratio) depends on peptide sequence on the sidewall (mean ± SD, *n*=3). f) E_11_*/E_11_ ratio increases with the number of hydrophobic residues (mean and individual values, *n*=6, 2, 4).

Encouraged by these promising results, we wanted to test next whether it is also possible to synthesize a whole peptide sequence on the Fmoc‐Phe‐SWCNT* **7**. Here, we chose a positively charged hexaarginine peptide to also evaluate its impact on SWCNT* solubility in aqueous environments (Figure 5 a). To evaluate the success of the SWCNT*‐peptide synthesis, neither the Kaiser test for free amines nor UV measurements after Fmoc cleavage could be used due to their insufficient sensitivities on the small scale of these experiments (*n*_SWCNT_=100 pmol in 1 mL solution). Thus, we decided to couple 5(6)‐CF to the *N*‐terminus before global deprotection of the arginine's side chains using a deprotection cocktail [75 % trifluoroacetic acid (TFA), 15 % dichloromethane (DCM), 5 % ddH_2_O, 5 % triisopropylsilane (TIS)]. This was followed by tip‐sonication in 1xPBS (3 min, 30 % amp, 4 °C) and centrifugation (16 100 g, 30 min) to remove insoluble SWCNTs. Figure 5 b shows colocalization of the NIR and the CF channels on the single‐nanotube level, indicating successful synthesis of SWCNT*‐F‐R_6_‐CF in contrast to the control (without **6**, see Figure S10) and retained optoelectronic properties after TFA deprotection. In fact, the negative control did not contain any SWCNTs, indicating increased solubility in aqueous environments by covalent peptide functionalization. However, due to the small number of defects (approximately one defect per 20 nm tube^30^) the SWCNT*‐F‐R_6_‐CF (**8**) did not display high solubility in water and therefore additional wrapping was used to increase the concentration and colloidal stability. In future studies, this aspect could be further evaluated with higher defect densities and/or longer peptide sequences.

As a next step we scaled up the SWCNT*‐based peptide synthesis and synthesized multiple SWCNT*‐peptide conjugates at the same time in a 96‐well format (equipped with 0.2 μm pore size filters, Figure 5 c). Again, the synthesis followed the same protocol as above, yet with smaller reaction volumes. Using this technique, we synthesized twelve different peptide sequences directly on NIR fluorescent carbon nanotubes. After side‐chain deprotection using the same deprotection cocktail as for SWCNT*‐F‐R_6_‐CF, the carbon nanotubes were redispersed in an aqueous 1 % DOC solution via tip‐sonication. While DOC leads to slightly red‐shifted emission compared to SDBS‐dispersed SWCNT*‐F‐Fmoc/SDBS (**7**),^59^, ^60^ both Figure 5 d and e show the impact of peptide sequence on the NIR fluorescence. For some sequences the original E_11_ peak was almost twice as intense as the E_11_^*^ peak—other sequences showed exactly the opposite behavior with the E_11_^*^ signal being 2.5‐fold stronger than the signal arising from the E_11_ transition (Vis/NIR absorbance and NIR fluorescence spectra in Figure S11). A closer evaluation of the sequence dependence of this fluorescence modulation shows that the E_11_^*^/E_11_ ratio decreases with an increasing number of hydrophobic residues in the peptide sequence attached to the defect responsible for exciton trapping (Figure 5 f). A similar effect was already observed by Kwon et al., who found changing E_11_^*^ emission wavelengths and E_11_^*^/E_11_ ratios for differently substituted (fluoro)alkyl/aryl sp^3^ defects.^32^ This interesting impact on the SWCNT photophysics could be attributed to the peptides folding differently on the SWCNT and changing the charge landscape through which the exciton diffuses or where it gets trapped, thus leading to enhanced E_11_^*^ fluorescence for less hydrophobic sequences. Different folding of peptides is known from noncovalent SWCNT/peptide hybrids.^61^ Furthermore, a comparison of the sequences 9–12 consisting of identical amino acids shows that not only the nature of the attached amino acids, but also their sequential arrangement is of high importance for the SWCNT* NIR fluorescence properties. These results demonstrate the possibilities of employing Fmoc‐protected phenylalanine defects for the growth of peptidic chains directly on the nanotube's sidewall and indicate that this method can not only be used for modulation of the SWCNTs’ fluorescence, but also to tailor their surface properties. This in turn could enable SWCNTs with enhanced cellular uptake/retention,^62^, ^63^ tailored molecular recognition motifs, and novel and more stable optical sensors operating in the NIR. Furthermore, the coupling of a second optically active molecule (fluorophore) via a peptide sequence to a SWCNT could serve as general design principle for molecular recognition and signal transduction. Similar to Förster resonance energy transfer (FRET), conformational changes upon binding to a target structure could affect the SWCNTs’ NIR fluorescence and enable novel fluorescent probes and labels.

## Conclusion

In summary, we introduced two new sp^3^ quantum defects in SWCNTs, which serve as anchors for the attachment of biomolecules. The versatility of this new functionalization platform was demonstrated by conjugation of a GFP‐binding nanobody as an example for a protein and the synthesis of peptides directly on the carbon nanotube surface. This new technique for covalent decoration of SWCNTs with biomolecules opens up great possibilities for applications in (bio)photonics, biosensing, and biomedicine.

## Conflict of interest

F. Opazo is shareholder of NanoTag Biotechnologies GmbH; all other authors declare no conflict of interest.

## Supporting information

As a service to our authors and readers, this journal provides supporting information supplied by the authors. Such materials are peer reviewed and may be re‐organized for online delivery, but are not copy‐edited or typeset. Technical support issues arising from supporting information (other than missing files) should be addressed to the authors.

Supplementary

## References

[1] S. Iijima , Nature 1991, 354, 56–58.

[2] S. Kruss , A. J. Hilmer , J. Zhang , N. F. Reuel , B. Mu , M. S. Strano , Adv. Drug Delivery Rev. 2013, 65, 1933–1950. 10.1016/j.addr.2013.07.015 23906934

[3] M. J. O′Connell , S. M. Bachilo , C. B. Huffman , V. C. Moore , M. S. Strano , E. H. Haroz , K. L. Rialon , P. J. Boul , W. H. Noon , C. Kittrell , et al., Science 2002, 297, 593–596. 12142535 10.1126/science.1072631

[4] S. M. Bachilo , M. S. Strano , C. Kittrell , R. H. Hauge , R. E. Smalley , R. B. Weisman , Science 2002, 298, 2361–2366. 12459549 10.1126/science.1078727

[5] C. Farrera , F. Torres Andón , N. Feliu , ACS Nano 2017, 11, 10637–10643. 29087693 10.1021/acsnano.7b06701

[6] K. Welsher , Z. Liu , S. P. Sherlock , J. T. Robinson , Z. Chen , D. Daranciang , H. Dai , Nat. Nanotechnol. 2009, 4, 773–780. 19893526 10.1038/nnano.2009.294 PMC2834239

[7] Z. Liu , S. M. Tabakman , Z. Chen , H. Dai , Nat. Protoc. 2009, 4, 1372–1381. 19730421 10.1038/nprot.2009.146 PMC2853228

[8] T. V. Galassi , P. V. Jena , J. Shah , G. Ao , E. Molitor , Y. Bram , A. Frankel , J. Park , J. Jessurun , D. S. Ory , et al., Sci. Transl. Med. 2018, 10, eaar2680. 30282694 10.1126/scitranslmed.aar2680 PMC6543545

[9] G. Pastorin , Pharm. Res. 2009, 26, 746–769. 19142717 10.1007/s11095-008-9811-0

[10] H. Huang , Q. Yuan , J. S. Shah , R. D. K. Misra , Adv. Drug Delivery Rev. 2011, 63, 1332–1339. 10.1016/j.addr.2011.04.001 21514336

[11] P. W. Barone , R. S. Parker , M. S. Strano , Anal. Chem. 2005, 77, 7556–7562. 16316162 10.1021/ac0511997

[12] A. J. Gillen , A. A. Boghossian , Front. Chem. 2019, 7, 13793–13713. 10.3389/fchem.2019.00612 PMC6763700 31616652

[13] F. A. Mann , Z. Lv , J. Grosshans , F. Opazo , S. Kruss , Angew. Chem. Int. Ed. 2019, 58, 11469–11473; 10.1002/anie.201904167 31112007

[14] F. A. Mann , N. Herrmann , D. Meyer , S. Kruss , Sensors 2017, 17, 1521. 10.3390/s17071521 PMC5539566 28657584

[15] M. Dinarvand , E. Neubert , D. Meyer , G. Selvaggio , F. A. Mann , L. Erpenbeck , S. Kruss , Nano Lett. 2019, 19, 6604–6611. 31418577 10.1021/acs.nanolett.9b02865

[16] S. Kruss , D. P. Salem , L. Vuković , B. Lima , E. Vander Ende , E. S. Boyden , M. S. Strano , Proc. Natl. Acad. Sci. USA 2017, 114, 1789–1794. 28179565 10.1073/pnas.1613541114 PMC5338365

[17] S. Kruss , M. P. Landry , E. Vander Ende , B. M. A. Lima , N. F. Reuel , J. Zhang , J. Nelson , B. Mu , A. Hilmer , M. Strano , J. Am. Chem. Soc. 2014, 136, 713–724. 24354436 10.1021/ja410433b

[18] H. Wu , R. Nißler , V. Morris , N. Herrmann , P. Hu , S.-J. Jeon , S. Kruss , J. P. Giraldo , Nano Lett. 2020, 20, 2432–2442. 32097014 10.1021/acs.nanolett.9b05159

[19] G. Bisker , J. Dong , H. D. Park , N. M. Iverson , J. Ahn , J. T. Nelson , M. P. Landry , S. Kruss , M. S. Strano , Nat. Commun. 2016, 7, 10241. 26742890 10.1038/ncomms10241 PMC4729864

[20] R. M. Williams , C. Lee , T. V. Galassi , J. D. Harvey , R. Leicher , M. Sirenko , M. A. Dorso , J. Shah , N. Olvera , F. Dao , et al., Sci. Adv. 2018, 4, eaaq1090. 29675469 10.1126/sciadv.aaq1090 PMC5906074

[21] M. Zheng , A. Jagota , M. S. Strano , A. P. Santos , P. Barone , S. G. Chou , B. A. Diner , M. S. Dresselhaus , R. S. Mclean , G. B. Onoa , et al., Science 2003, 302, 1545–1548. 14645843 10.1126/science.1091911

[22] R. Nißler , F. A. Mann , P. Chaturvedi , J. Horlebein , D. Meyer , L. Vuković , S. Kruss , J. Phys. Chem. C 2019, 123, 4837–4847.

[23] A. J. Gillen , J. Kupis-Rozmysłowicz , C. Gigli , N. Schuergers , A. A. Boghossian , J. Phys. Chem. Lett. 2018, 9, 4336–4343. 30004705 10.1021/acs.jpclett.8b01879

[24] E. Polo , S. Kruss , J. Phys. Chem. C 2016, 120, 3061–3070.

[25] A. Hirsch , Angew. Chem. Int. Ed. 2002, 41, 1853–1859; 10.1002/1521-3773(20020603)41:11<1853::aid-anie1853>3.0.co;2-n 19750614

[26] A. Setaro , M. Adeli , M. Glaeske , D. Przyrembel , T. Bisswanger , G. Gordeev , F. Maschietto , A. Faghani , B. Paulus , M. Weinelt , et al., Nat. Commun. 2017, 8, 838–837. 28134240 10.1038/ncomms14281 PMC5290266

[27] A. G. Godin , A. Setaro , M. Gandil , R. Haag , M. Adeli , S. Reich , L. Cognet , Sci. Adv. 2019, 5, eaax1166. 31799400 10.1126/sciadv.aax1166 PMC6868679

[28] L. Chio , R. L. Pinals , A. Murali , N. S. Goh , M. P. Landry , Adv. Funct. Mater. 2020, 30, 1910556–1910558.

[29] S. Ghosh , S. M. Bachilo , R. A. Simonette , K. M. Beckingham , R. B. Weisman , Science 2010, 330, 1656–1659. 21109631 10.1126/science.1196382

[30] Y. M. Piao , B. Meany , L. R. Powell , N. Valley , H. Kwon , G. C. Schatz , Y. H. Wang , Nat. Chem. 2013, 5, 840–845. 24056340 10.1038/nchem.1711

[31] F. J. Berger , J. Lüttgens , T. Nowack , T. Kutsch , S. Lindenthal , L. Kistner , C. C. Müller , L. M. Bongartz , V. A. Lumsargis , Y. Zakharko , et al., ACS Nano 2019, 13, 9259–9269. 31381849 10.1021/acsnano.9b03792 PMC6716210

[32] H. Kwon , M. Furmanchuk , M. Kim , B. Meany , Y. Guo , G. C. Schatz , Y. Wang , J. Am. Chem. Soc. 2016, 138, 6878–6885. 27159413 10.1021/jacs.6b03618 PMC4915342

[33] Y. Miyauchi , M. Iwamura , S. Mouri , T. Kawazoe , M. Ohtsu , K. Matsuda , Nat. Photonics 2013, 7, 715–719.

[34] M. Kim , X. Wu , G. Ao , X. He , H. Kwon , N. F. Hartmann , M. Zheng , S. K. Doom , Y. Wang , Chem 2018, 4, 2180–2191. 31763495 10.1016/j.chempr.2018.06.013 PMC6874404

[35] C. F. Chiu , W. A. Saidi , V. E. Kagan , A. Star , J. Am. Chem. Soc. 2017, 139, 4859–4865. 28288512 10.1021/jacs.7b00390 PMC6540762

[36] P. Clément , P. Trinchera , K. Cervantes Salguero , Q. Ye , C. R. Jones , M. Palma , ChemPlusChem 2019, 84, 1235–1238. 31944048 10.1002/cplu.201900151

[37] H. Kwon , M. Kim , B. Meany , Y. Piao , L. R. Powell , Y. Wang , J. Phys. Chem. C 2015, 119, 3733–3739.

[38] T. Shiraki , H. Onitsuka , T. Shiraishi , N. Nakashima , Chem. Commun. 2016, 52, 12972–12975. 10.1039/c6cc07287a 27747344

[39] N. Danné , M. Kim , A. G. Godin , H. Kwon , Z. Gao , X. Wu , N. F. Hartmann , S. K. Doorn , B. Lounis , Y. Wang , et al., ACS Nano 2018, 12, 6059–6065. 29889499 10.1021/acsnano.8b02307

[40] X. He , H. Htoon , S. K. Doorn , W. H. P. Pernice , F. Pyatkov , R. Krupke , A. Jeantet , Y. Chassagneux , C. Voisin , Nat. Mater. 2018, 17, 663–670. 29915427 10.1038/s41563-018-0109-2

[41] X. He , N. F. Hartmann , X. Ma , Y. Kim , R. Ihly , J. L. Blackburn , W. Gao , J. Kono , Y. Yomogida , A. Hirano , et al., Nat. Photonics 2017, 11, 577.

[42] A. K. Mandal , X. Wu , J. S. Ferreira , M. Kim , L. R. Powell , H. Kwon , L. Groc , Y. Wang , L. Cognet , Sci. Rep. 2020, 10, 5286. 32210295 10.1038/s41598-020-62201-w PMC7093457

[43] C.-W. Lin , S. M. Bachilo , Y. Zheng , U. Tsedev , S. Huang , R. B. Weisman , A. M. Belcher , Nat. Commun. 2019, 10, 2874. 31253811 10.1038/s41467-019-10917-3 PMC6599008

[44] A. H. Brozena , M. Kim , L. R. Powell , Y. Wang , Nat. Rev. Chem. 2019, 3, 375–392. 32789186 10.1038/s41570-019-0103-5 PMC7418925

[45] J. Helma , M. C. Cardoso , S. Muyldermans , H. Leonhardt , J. Cell Biol. 2015, 209, 633–644. 26056137 10.1083/jcb.201409074 PMC4460151

[46] C. Hamers-Casterman , T. Atarhouch , S. Muyldermans , G. Robinson , C. Hammers , E. B. Songa , N. Bendahman , R. Hammers , Nature 1993, 363, 446–448. 8502296 10.1038/363446a0

[47] J. Ries , C. Kaplan , E. Platonova , H. Eghlidi , H. Ewers , Nat. Methods 2012, 9, 582–584. 22543348 10.1038/nmeth.1991

[48] M. Mikhaylova , B. M. C. Cloin , K. Finan , R. Van Den Berg , J. Teeuw , M. M. Kijanka , M. Sokolowski , E. A. Katrukha , M. Maidorn , F. Opazo , et al., Nat. Commun. 2015, 6, 7933. 26260773 10.1038/ncomms8933 PMC4918323

[49] S. Sograte-Idrissi , N. Oleksiievets , S. Isbaner , M. Eggert-Martinez , J. Enderlein , R. Tsukanov , F. Opazo , Cells 2019, 8, 48. 10.3390/cells8010048 PMC6357156 30646582

[50] H. D. Herce , D. Schumacher , A. F. L. Schneider , A. K. Ludwig , F. A. Mann , M. Fillies , M.-A. Kasper , S. Reinke , E. Krause , H. Leonhardt , et al., Nat. Chem. 2017, 9, 762–771. 28754949 10.1038/nchem.2811

[51] S. Kabatas , P. Agüi-Gonzalez , K. A. Saal , S. Jähne , F. Opazo , S. O. Rizzoli , N. T. N. Phan , Angew. Chem. Int. Ed. 2019, 58, 3438–3443; 10.1002/anie.201812032 PMC6593772 30614604

[52] J. M. J. M. Ravasco , H. Faustino , A. Trindade , P. M. P. Gois , Chem. Eur. J. 2019, 25, 43–59. 30095185 10.1002/chem.201803174

[53] G. Grigoryan , Y. H. Kim , R. Acharya , K. Axelrod , R. M. Jain , L. Willis , M. Drndic , J. M. Kikkawa , W. F. DeGrado , Science 2011, 332, 1071–1076. 21617073 10.1126/science.1198841 PMC3264056

[54] F. A. Mann , J. Horlebein , N. F. Meyer , D. Meyer , F. Thomas , S. Kruss , Chem. Eur. J. 2018, 24, 12241–12245. 29488660 10.1002/chem.201800993

[55] G. R. Dieckmann , A. B. Dalton , P. A. Johnson , J. Razal , J. Chen , G. M. Giordano , E. Muñoz , I. H. Musselman , R. H. Baughman , R. K. Draper , J. Am. Chem. Soc. 2003, 125, 1770–1777. 12580602 10.1021/ja029084x

[56] E. Polo , T. T. Nitka , E. Neubert , L. Erpenbeck , L. Vuković , S. Kruss , ACS Appl. Mater. Interfaces 2018, 10, 17693–17703. 29708725 10.1021/acsami.8b04373

[57] A. Bianco , K. Kostarelos , C. D. Partidos , M. Prato , Chem. Commun. 2005, 571–577. 10.1039/b410943k 15672140

[58] D. Pantarotto , C. D. Partidos , R. Graff , J. Hoebeke , J.-P. Briand , M. Prato , A. Bianco , J. Am. Chem. Soc. 2003, 125, 6160–6164. 12785847 10.1021/ja034342r

[59] R. Haggenmueller , S. S. Rahatekar , J. A. Fagan , J. Chun , M. L. Becker , R. R. Naik , T. Krauss , L. Carlson , J. F. Kadla , P. C. Trulove , et al., Langmuir 2008, 24, 5070–5078. 18442227 10.1021/la703008r

[60] T. Shiraki , Y. Niidome , F. Toshimitsu , T. Shiraishi , T. Shiga , B. Yu , T. Fujigaya , Chem. Commun. 2019, 55, 3662–3665. 10.1039/c9cc00829b 30855053

[61] D. A. Heller , G. W. Pratt , J. Zhang , N. Nair , A. J. Hansborough , A. A. Boghossian , N. F. Reuel , P. W. Barone , M. S. Strano , Proc. Natl. Acad. Sci. USA 2011, 108, 8544–8549. 21555544 10.1073/pnas.1005512108 PMC3102399

[62] M. Gravely , M. M. Safaee , D. Roxbury , Nano Lett. 2019, 19, 6203—6212. 31424226 10.1021/acs.nanolett.9b02267 PMC7199458

[63] D. Meyer , S. Telele , A. Zelená , A. J. Gillen , A. Antonucci , E. Neubert , R. Nißler , F. A. Mann , L. Erpenbeck , A. A. Boghossian , et al., Nanoscale 2020, 2, 751. 10.1039/d0nr00864h 32286598

