# Supplementary material for: Quantum Defects as a Toolbox for the Covalent Functionalization of Carbon Nanotubes with Peptides and Proteins
Source: Angew Chem Int Ed Engl. 2020 Jul 13;59(40):17732–8. doi: 10.1002/anie.202003825 (PMC7540668; doi:10.1002/anie.202003825)

Angewandte Chemie
Eine Zeitschrift der Gesellschaft Deutscher Chemiker

Supporting Information

Quantum Defects as a Toolbox for the Covalent Functionalization of Carbon Nanotubes with Peptides and Proteins

*Florian A. Mann, Niklas Herrmann, Felipe Opazo, and Sebastian Kruss**

anie_202003825_sm_miscellaneous_information.pdf

Table of contents

1. Experimental Procedures 2

1.1. General Information 2

1.2. Chemical synthesis 3

1.2.1. 4-(*N*-maleimido)phenyldiazonium tetrafluoroborate (2) 3

Fmoc-L-4-diazonium-phenylalanine tetrafluoroborate (6) 3

1.3. Carbon nanotube reactions 4

1.3.1. Synthesis of SWCNT*-Ph-Mal (3) 4

1.3.2. Synthesis of SWCNT*-Ph-GBP (5) 4

1.3.2.1. Sequential reaction 4

1.3.2.2. One-step reaction 4

1.3.3. Synthesis of SWCNT*-Phe-Fmoc (7) 4

1.3.4. Synthesis of SWCNT*-Phe-5(6)-CF (10) 5

1.3.3 Synthesis of SWCNT*-Phe-R_6-CF (11) 5

1.3.4 96-well synthesis of SWCNT*-Peptides 5

1.4 *In vitro* GFP-binding assay 6

1.5 VIS/NIR fluorescence microscopy of SWCNT*-F-CF, SWCNT*-F-R_6-CF and the corresponding controls 6

1.6 VIS-fluorescence spectroscopy of SWCNT*-F-CF 6

2. Results and Discussion 7

3. References 14

4. Author Contributions 14

5. NMR spectra of diazonium salts 2 and 6 15

5.1. 4-(*N*-maleimido)phenyldiazonium tetrafluoroborate (2) 15

5.2. Fmoc-L-4-diazonium-phenylalanine tetrafluoroborate (6) 19

1. Experimental Procedures

1.1. General Information

NMR spectra were recorded with a Bruker™ Avance III HD 300 device (Bruker Corp., USA), equipped with a 5 mm probe. For a measurement, approx. 15 mg of substance were dissolved in approx. 600 µL of the deuterated solvent stated and transferred to a standard glass NMR tube (d = 5 mm). The chemical shifts are reported in ppm relative to the residual solvent peak(s). Analysis was performed within MestReNova™ 10.

ESI-TOF-MS measurements of diazonium salts were performed on a Bruker™ micrOTOF ESI-TOF-MS using the non-deuterated form of the solvent also used for NMR measurements.

VIS-fluorescence spectra were acquired between 500 and 600 nm using an excitation wavelength of 490 nm on a FluoroMax-4 spectrofluorometer (Horiba Scientific, Japan).

Reagents and solvents were, unless stated otherwise, of synthesis grade quality and used without further purification. Fmoc-protected amino acids were purchased either from IRIS Biotech (Germany) or Novabiochem (Germany).

(6,5)-chirality enriched single-walled carbon nanotubes (SWCNTs, Product No.: 773735) were acquired from Sigma Aldrich (Darmstadt, Germany).

SWCNT/SDBS stock solutions were generated by tip sonication of a SWCNT/SDBS (1%) suspension for 15 minutes (Fisher Scientific™ Model 120 Sonic Dismembrator, 30% amplitude, 36 W) followed by centrifugation (2x, 16100g). Only the supernatant (top 80%) was used for further studies.

Experiments and measurements were, unless otherwise stated, performed in phosphate buffered saline (PBS, 8.2 mM Na2HPO4, 1.8 mM K2HPO4, 137 mM NaCl, 2.7 mM KCl, pH 7.4).

Protein concentrations were determined *via* absorbance measurements at 280 nm with a NanoDrop 2000™ spectrophotometer (ThermoFisher Scientific Inc., USA) using the extinction coefficient of the respective protein at 280 nm. The mean value of at least three independent measurements was taken for concentration calculation.

VIS/NIR absorbance spectroscopy was conducted on a JASCO V-670 (Spectra Manager Software) using a 10 mm-path cuvette. Spectra were acquired using a scan speed of 1000 $nm \cdot min^{-1}$, a data interval of 0.5 nm and a UV/vis and nIR bandwidth of 2 nm and 4 nm, respectively.
SWCNT concentration was estimated using the maximal absorbance at approx. 990 nm using the molar extinction coefficient determined by Schöppler *et al*.[1]

NIR fluorescence spectroscopy was conducted in glass-bottom 96-well plates *via* excitation at 561 nm using a gem-561 laser (LaserQuantum™, Germany) at 100 mW excitation power and fluorescence spectra were recorded in the range between 850 and 1250 nm using a Shamrock 193i spectrograph (Andor Technology Ltd., Belfast, Northern Ireland) coupled to an Olympus IX73 microscope and an exposure time of 1 s, a slit width of 500 µm and an Andor iDus InGaAs 491 array NIR detector

2D-excitation-emission maps were recorded in glass-bottom 96-well plates *via* excitation between 400 and 800 nm using a 300 W Xe-lamp guided through a monochromator (LOT, Germany) and detection between 850 and 1250 nm using a Shamrock 193i spectrograph (Andor Technology Ltd., Belfast, Northern Ireland) coupled to an Olympus IX73 microscope and an exposure time of 5 s, a slit width of 500 µm and an Andor iDus InGaAs 491 array NIR detector.

Atomic Force Microscopy (AFM) was conducted in intermittent-contact mode (scan rate = 0.5 Hz, 512 lines) using an Asylum Research MFP-3D Infinity® instrument equipped with rectangular cantilevers (Opus, MikroMasch Europe, Al-coating, tetrahedral tip, ν_{res} = 300 kHz, k = 26 $N \cdot m^{-1}$). Freshly cleaved muscovite mica was incubated with a poly-L-lysine (PLL, 0.1 $mg \cdot mL^{-1}$, 10 min) solution. After washing with MilliQ water, the coated mica was incubated with 10 µL of the sample solution for another 10 minutes followed by repeated washing of the surface with MilliQ water and drying using a N_2-stream before sample measurement.
Analysis of the acquired images was performed via the open-source software Gwyddion.

VIS-NIR-fluorescence microscopy was carried out under 561 nm-laser excitation (Cobolt Jive laser, Cobolt AB, Solna, Sweden, $P_{max.}$ = 500 mW) on an Olympus IX53 microscope equipped with a 100x oil-immersion objective (Olympus 100x UPLSAPO 100XS, NA = 1.35). Detection of the near-infrared photoluminescence was carried using a Xenics Cheetah-640-TE3 NIR camera (Xenics,

Heverlee, Belgium), while the VIS-fluorescence was detected with an Andor Zyla 5.5 sCMOS camera (Andor Technology Ltd., Belfast, Northern Ireland).

1.2. Chemical synthesis

1.2.1. 4-(*N*-maleimido)phenyldiazonium tetrafluoroborate (2)

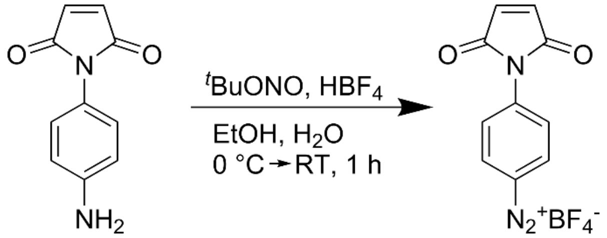

4-(*N*-Maleimido)phenyldiazonium tetrafluoroborate was synthesized using an optimized procedure based on the general procedure given in [2]. In a glass snap-cap vial, 4-(*N*-aminophenyl)maleimide (95.1 mg, 505 µmol, 1.0 eq.) was dissolved in ethanol (500 µL). Tetrafluoroboronic acid (50% aqueous solution, 125 µL, 175 mg, 996 µmol, 2.0 eq.) was added and the resulting red suspension was cooled to 0 °C in an ice/water bath. Under magnetic stirring, *tert*-butyl nitrite (135 µL, 117 mg, 1.13 mmol, 2.2 eq.) was added dropwise *via* syringe. The resulting greyish suspension was stirred for 1 h at room temperature. Diethyl ether (1 mL) was added and the resulting grey suspension was transferred to a polypropylene vial and centrifuged (2 min at 16100 g). The yellow supernatant was decanted off and the remaining solid was suspended in diethyl ether (1 mL). The centrifugation-decantation-suspension washing cycle was repeated three times before the resulting solid was dried under reduced pressure at room temperature to yield a slightly yellow solid.
While standard precautions were taken during synthesis and handling of the herein presented diazonium salts, no decomposition was observed even over several weeks.

^{1}H-NMR (300 MHz**, CD_3CN):** δ (ppm) = 8.54-8.60 (m, 2*H*, $H_{2,6}$), 8.09-8.15 (m, 2*H*, $H_{3,5}$), 7.08 (s, 2*H*, $H_{maleimide}$).

^{11}B-NMR (96 MHz**, CD_3CN):** δ (ppm) = −1.16 (s).

^{19}F-NMR (282 MHz**, CD_3CN):** δ (ppm) = −151.48 (s), −151.54 (s) (two signals due to the two NMR-active boron isotopomers).

^{13}C-NMR (75 MHz**, CD_3CN):** δ (ppm) = 169.4, 144.8, 136.5, 134.7, 127.4, 111.2.

The NMR data is in good agreement with literature data. [3]

HRMS (ESI (pos.) [*m/z*]**):** calculated ($C_{10}H_6N_3O_2$ [M^+]): 200.0455, found: 200.0448; calculated ($C_{10}H_6NO_2$ [$M-N_2$]$^+$): 172.0393, found: 172.0389; ($C_{11}H_{10}NO_3$ [$M-N_2+MeOH$]$^+$): 204.0645, found: 204.0655.

Fmoc-L-4-diazonium-phenylalanine tetrafluoroborate (6)

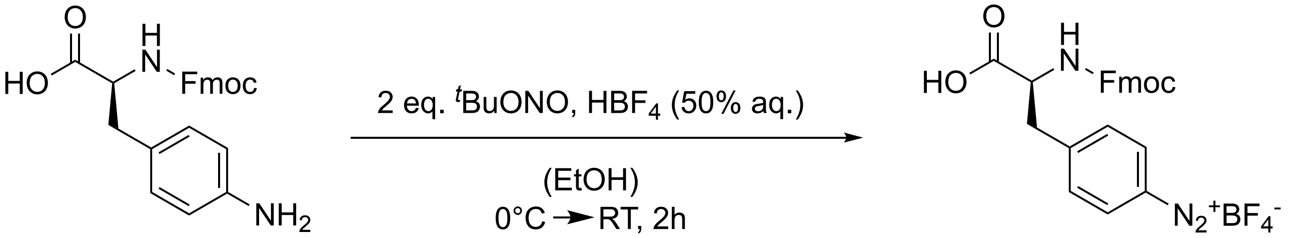

Fmoc-L-4-diazonium-phenylalanine tetrafluoroborate was synthesized using an optimized procedure based on the general procedure given in [2]. Fmoc-L-4-aminophenylalanine (0.2 g, 0.497 mmol) was dissolved in 500 µL ethanol in a 5 mL glass scintillation vial. Next, approx. 125 µL HBF_4 (50% aqueous,) were added dropwise. The solution was placed in an ice bath and stirred constantly while 135 µL ^{t}Bu-ONO (2 eq., 1.1 mmol, 117 mg) were added dropwise over the course of 30 min. Next, the solution was stirred for two hours at rt. After the reaction was completed, a white/slightly yellow precipitate was observed and transferred to a 2 mL Eppendorf tube using 1 mL diethyl ether. The precipitate was centrifuged at 16100g for five minutes and the supernatant discarded. This process was repeated five times and the crude product was dried under reduced pressure at room temperature.

While standard precautions were taken during synthesis and handling of the herein presented diazonium salts, no decomposition was observed even over several weeks.

^{1}H-NMR (300 MHz**, CD_3CN):** δ (ppm) = 8.35 (d, *J* = 8.5 Hz, 2H), 7.84 (d, *J* = 7.5 Hz, 2H), 7.76 (d, *J* = 8.4 Hz, 2H), 7.61 (dd, *J* = 7.5, 4.6 Hz, 2H), 7.43 (t, *J* = 7.5 Hz, 2H), 7.35 (t, *J* = 7.5 Hz, 2H), 6.13 (d, *J* = 8.7 Hz, 1H), 4.53 (s, 1H), 4.30 (d, *J* = 6.2 Hz, 2H), 4.18 (t, *J* = 6.8 Hz, 1H), 3.50 – 3.41 (m, 1H), 3.27 – 3.16 (m, 1H).

^{11}B-NMR (96 MHz**, CD_3CN):** δ (ppm) = −1.15 (s).

^{19}F-NMR (282 MHz**, CD_3CN):** δ (ppm) = −151.47 (s), −151.53 (s) (two signals due to the two NMR-active boron isotopomers).

HRMS (ESI (pos.) [*m/z*]**):** calculated ($C_{24}H_{21}NO_4$ $[M-N_2]^+$): 386.1392, found: 386.1387.

1.3. Carbon nanotube reactions

1.3.1. Synthesis of SWCNT*-Ph-Mal (3)

A (6,5)-chirality enriched SWCNT solution (**1,** 180 µL of a 10 nmol L^{-1} solution in 1% SDBS) was mixed with an aqueous solution of 4-(*N*-maleimido)phenyldiazonium tetrafluoroborate (**2,** 20 µL, 1 mmol L^{-1}) and irradiated in a 96-well plate using a Lumidox™ (Analytical Sales & Services, Inc., Flanders, NJ, USA) 96 green LED array with an LED current of 25 mA. After reaction control using nIR fluorescence spectroscopy, the reaction mixtures were transferred to spin filters (Vivaspin™ 500, MWCO = 100 kDa, *v* = 500 µL, Sartorius, Göttingen, Germany) and centrifuged (12000 g, RT, final volume = approx. 50 µL). The SWCNT* precipitated on the filter's membrane were washed with water (5× 450 µL), each time followed by a centrifugation step, and finally resuspended in a solution of the chosen surfactant (either 1% SDBS or 2 mg/mL 1,2-distearoyl-*sn*-glycero-3-phosphoethanolamine-N-[methoxy(polyethylene glycol)-5000] (ammonium salt) [PL-PEG5000]) by scraping off the filter and repeated pipetting. For redispersion, those suspensions were tip-sonicated (30% amplitude, 36 W, 5 min, 0 °C), centrifuged (16100g, 30 min) and the supernatant used for further studies.

1.3.2. Synthesis of SWCNT*-Ph-GBP (5)

1.3.2.1. Sequential reaction

A (6,5)-chirality enriched SWCNT solution (**1,** 180 µL of a 10 nmol L^{-1} solution in 1% SDBS) was mixed with an aqueous solution of 4-(*N*-maleimido)phenyldiazonium tetrafluoroborate (**2,** 20 µL, 1 mmol L^{-1}) and 6.4 µL of a 156 µM GBP-Nanobody (**4**, previously reduced on ice using TCEP [5 mM] for 30 minutes) solution in a 96-well plate. The mixed solution was irradiated using a Lumidox™ (Analytical Sales & Services, Inc., Flanders, NJ, USA) 96 green LED array with an LED current of 25 mA for 15 minutes followed by removal of the excess MalPh-Dz **2** using spin-filtration (300 kDa MWCO) and subsequent resuspension of the MalPh-SWCNT* **3** in 1x phosphate-buffered saline (PBS, pH 7.4). 500 eq. (~25 eq. with respect to introduced maleimides) of the nanobody **4** were added and the solution left shaking gently for 16 hours at room temperature. To remove excess unreacted nanobody, 300 kDa-MWCO spin filtration was carried out (5x washing with 1x PBS) followed by redispersion using PL-PEG5000 (2 mg·mL^{-1}).

1.3.2.2. One-step reaction

A (6,5)-chirality enriched SWCNT solution (**1,** 180 µL of a 10 nmol L^{-1} solution in 1% SDBS) was mixed with an aqueous solution of 4-(*N*-maleimido)phenyldiazonium tetrafluoroborate (**2,** 20 µL, 1 mmol L^{-1}) and 6.4 µL of a 156 µM GBP-Nanobody (**4**, previously reduced on ice using TCEP [5 mM] for 30 minutes) solution in a 96-well plate. The mixed solution was irradiated using a Lumidox™ (Analytical Sales & Services, Inc., Flanders, NJ, USA) 96 green LED array with an LED current of 25 mA for 30 minutes and subsequently transferred to a microcentrifuge tube and left shaking gently for 15 hours at room temperature. To remove excess unreacted nanobody, 300 kDa-MWCO spin filtration was carried out (5x washing with 1x PBS) followed by redispersion using PL-PEG5000 (2 mg·mL^{-1}).

1.3.3. Synthesis of SWCNT*-Phe-Fmoc (7)

To generate a SWCNT-SDBS dispersion (1% SDBS), 500 µL of an aqueous 2% SDBS solution were added to 500 µL of a 2 mg/mL SWCNT/water suspension. This solution was tip-sonicated for 15 minutes (Fisher Scientific™ Model 120 Sonic Dismembrator, 30% amplitude, 36 W) followed by centrifugation (2x, 16100g) and only the supernatant (top 80%) used for

further studies. In the next step, the supernatant was diluted using 1%SDBS to yield a 10 nM solution (**1,** see SWCNT concentration determination, section 1.1). In a 96well-plate, 180 µL of this solution were added to 15 wells followed by the addition of different concentrations of Fmoc-Phe-$N_2^+BF_4^-$ (**6**, 0 nM-100 µM). After careful mixing via repeating pipetting, the solutions were irradiated using a green 96-LED-array irradiator (Lumidox™, Analytical Sales & Services, Inc., Flanders, NJ, USA) at 25 mA for 15 minutes to yield solutions of **7**. The solutions were analyzed by nIR fluorescence spectroscopy for the degree of defect-introduction. The excess diazonium salt **6** was removed via repeated 300kDa-cut-off spin filtration (5x 600 µL ddH_2O washing) and the remaining SWCNT* were resuspended in 500 µL ddH_2O. In the last step, supernatant and pellet were then separated by centrifugation (16100g, 15 minutes) and the pellet containing SWCNT*-Phe-Fmoc **7** was used for downstream experiments.

1.3.4. Synthesis of SWCNT*-Phe-5(6)-CF (10)

The solutions of three wells from the Fmoc-Phe defect introduction process (v = 600 µL, approx. 5.4 pmol SWCNT*-Phe-Fmoc **7**) were added to a frit (20 µm pore size)-equipped syringe reactor (v = 2 mL), mixed with 1 mL EtOH for SWCNT precipitation and subsequent washing and removal of residual SDBS surfactant and excess Fmoc-Phe-Dz (5x 1 mL dH_2O, 3x DMF, 3x DCM, 3x DMF). Deprotection of the Fmoc-group was achieved via incubation with 200 µL of 20% piperidine/dimethylformamide (DMF, 2x 15 min). Excess reagents were removed by repeated washing with DMF/dichloromethane (DCM)/DMF (3x 1 mL). Subsequent amide coupling to 5(6)-carboxyfluorescein (CF) was carried out in the dark for 30 minutes at room temperature after the addition of 1.88 mg 5(6)-CF (5 µmol), 1.9 mg HATU (5 µmol) and 1.74 µL N,N-Diisopropylethylamine (DIPEA, 10 µmol) in 50 µL DMF. Excess reagents were removed by repeated washing with DMF/DCM/DMF (3x 1 mL). The crude product **10** was scraped off the frit using 50 µL DMF, transferred to a 1.5 mL microcentrifuge tube and centrifuged at 16100 g (15 min). The pellet was resuspended in 100 µL of a 1% SDBS solution and ultrasonicated (3 min, RT, 30% amplitude) followed by centrifugation (15 min, 16100g) and separation of pellet and supernatant. The latter was used for fluorescence microscopy and spectroscopy applications.

1.3.3 Synthesis of SWCNT*-Phe-R_6-CF (11)

The solutions of 20 wells from the Fmoc-Phe defect introduction process (v = 4 mL, approx. 36 pmol SWCNT*-Phe-Fmoc **7**) were pooled, mixed with 6 mL EtOH for SWCNT precipitation in a glass vial and the suspension subsequently transferred to a frit (20 µm pore size)-equipped syringe reactor (v = 2 mL). Residual SDBS surfactant and excess Fmoc-Phe-Dz were removed *via* washing (5x 1 mL dH_2O, 3x DMF, 3x DCM, 3x DMF). Deprotection of the Fmoc-group was achieved via incubation with 200 µL of 20% piperidine/DMF (2x 15 min). Fmoc-based solid-phase peptide synthesis (SPPS) couplings to Fmoc-Arg(Pbf)-OH (11.7 mg, 18 µmol) and 5(6)-CF (6.8 mg, 18 µmol) were carried out for 30 minutes at room temperature with 6.8 mg 1-[Bis(dimethylamino)methylene]-1*H*-1,2,3-triazolo[4,5-b]pyridinium 3-oxide hexafluorophosphate (HATU, 18 µmol) and 6.3 µL DIPEA (36 µmol) in 100 µL DMF. Excess reagents were removed by repeated washing with DMF/DCM/DMF (3x 1 mL). After 5(6)-CF coupling and washing, the side-chain protecting groups ((2,2,4,6,7-pentamethyldihydrobenzofuran-5-sulfonyl), Pbf) were cleaved upon gentle shaking with the cleavage cocktail (75% TFA/20% DCM/2.5% H_2O/2.5% TIS, v_{total} = 500 µL) for one hour. Subsequently, the crude product **5** was washed again with DMF and 10x DCM and then scraped off the frit using 100 µL H_2O, transferred to a 1.5 mL microcentrifuge tube and stored in the dark at 4°C. For fluorescence microscopy and spectroscopy applications, the product **11** in 100 µL H_2O was submitted to ultrasonication (5 min, 30% amp, 4°C) followed by separation of supernatant and pellet by centrifugation (16100g, 30 min). The supernatant was used for fluorescence microscopy and spectroscopy applications.

1.3.4 96-well synthesis of SWCNT*-Peptides

Approx. 30 pmol SWCNT*-Phe-Fmoc (*c* = 10 nM, *V* = 3 mL) were added to and filtered through individual wells of a 96-well plate equipped with 0.2 µm pore size filters (Chromafil Multi 96 plate with PTFE filters, Macherey-Nagel, Germany). The samples were washed (with 5x H_2O, 3x DMF, 3x DCM, 3x DMF)) followed by standard Fmoc/OtBu SPPS (2x15min Fmoc deprotection [20% Piperidine/DMF], 30min amino acid coupling [13.5 µmol amino acid/HATU, 27 µmol DIPEA]) and final deprotection of side-chain protecting groups using the deprotection cocktail (375 µL TFA, 100 µL DCM, 12.5 µL TIS, 12.5 µL H_2O, 60 min). All steps were carried out under mild agitation of the 96-well plate on a shaker (150 rpm). After final deprotection, the SWCNT samples were again washed (using 3xDCM, 3xDMF, 10xDCM) and then resuspended using 600 µL H_2O and transferred to 1.5 mL centrifuge tubes. Next, the samples were spun down (16100g, 30 min), resuspended in 200 µL 1% sodium deoxycholate (DOC) solution and submitted to tip-sonication (5 min, 4°C, 30% amplitude). After centrifugation, the top 80% of the supernatant were again transferred to fresh microcentrifuge tubes and used for absorbance/fluorescence spectroscopy applications.

The following Fmoc-protected amino acids were used:

Fmoc-Asn(Trt)-OH	Fmoc-Gly-OH	Fmoc-Tyr(tBu)-OH	Fmoc-Asp(OtBu)-OH
Fmoc-His(Trt)-OH	Fmoc-Gln(Trt)-OH	Fmoc-Ser(tBu)-OH	Fmoc-Glu(OtBu)-OH
Fmoc-Leu-OH	Fmoc-Lys(Boc)-OH	Fmoc-Val-OH	Fmoc-Phe-OH
Fmoc-Ile-OH	Fmoc-Ala-OH	Fmoc-Thr(tBu)-OH	

1.4 In vitro GFP-binding assay

Based on a previously created silicon master, patterned PDMS stamps were created. Next, a 2 µM GFP solution was added to the stamp, incubated for 10 minutes and the stamp subsequently rinsed with 1x PBS. In the next step, the PDMS stamp was transferred to a glass-bottom 96well-plate (previously coated with poly-L-lysine [0.1 mg mL^{-1}, 10 min] and incubated for 30 minutes followed by washing (1xPBS, 0.3% TritonX100, 3x). In the next step, the GFP-modified surface was passivated using a 5% w/v BSA solution (15 min) and subsequently washed again (1xPBS, 0.3% TritonX100, 3x). Finally, the conjugate **5** and its corresponding controls were added to the wells and incubated for 30 minutes, followed again by washing (1xPBS, 0.3% TritonX100, 3x) and addition of 200 µL of 1x PBS before imaging.

1.5 VIS/NIR fluorescence microscopy of SWCNT*-F-CF, SWCNT*-F-R_6-CF and the corresponding controls

Each 5 µL of the respective solutions were immobilized on a glass-coverslip *via* spin-coating (RT, 1000 rpm). Mounted on a glass-cover-slide, CF/nIR imaging was carried out on an Olympus IX53 microscope with a 100x oil-immersion objective (Olympus 100x UPLSAPO 100XS, NA=1.35) using 561 nm excitation by a Cobolt Jive™ laser (Cobolt AB, Solna, Sweden, 200 mW). CF fluorophores were excited by a xCite 120Q lamp (Excelitas Technologies, USA) using the lowest intensity/iris step. Detection of the near-infrared photoluminescence was carried using a Xenics® Cheetah SWIR camera (Xenics, Heverlee, Belgium, t_{int} = 1 s), whereas CF fluorescence was observed by an Andor Zyla 5.5 sCMOS camera (Andor Technology Ltd., Belfast, Northern Ireland, t_{int} = 0.1 s). Image overlay was conducted using a custom Python script.

1.6 VIS-fluorescence spectroscopy of SWCNT*-F-CF

The samples were diluted 200-fold to a final volume of 1000 µL and the fluorescence spectra recorded as described in section 1.1.

2. Results and Discussion

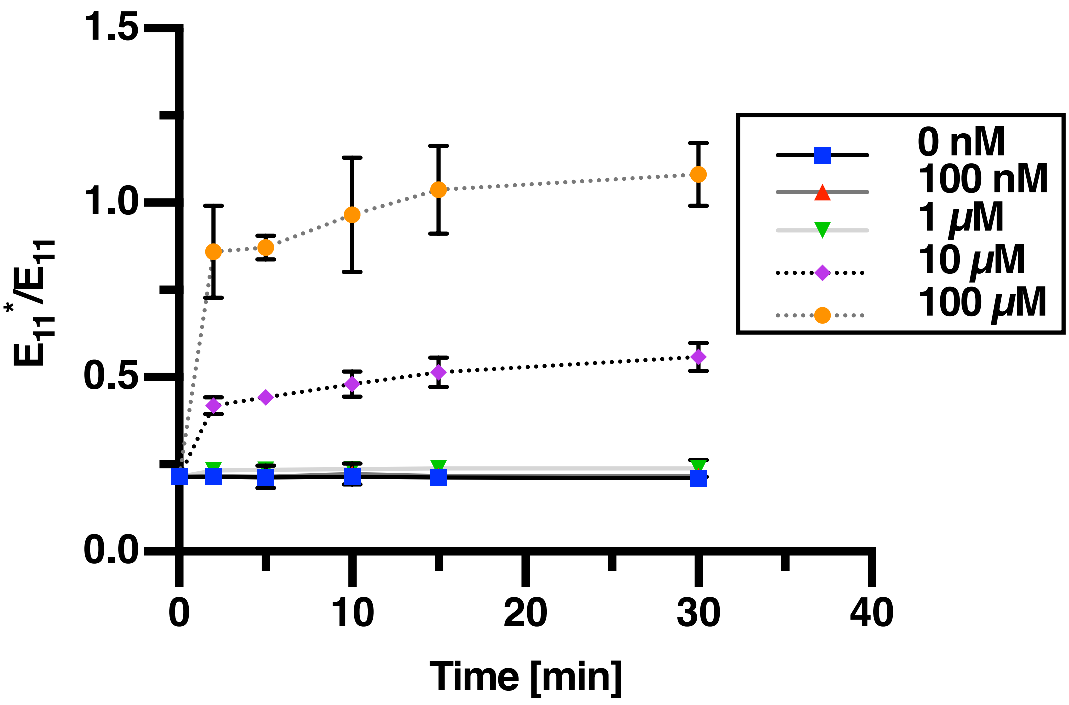

Figure S1. Evolution of the E_{11}^* emission peak over time when irradiated with a green 96-LED-array and different concentrations of MalPh diazonium salt **2** (*n* = 3, mean ± SD). The defect introduction was carried out in triplicates for each MalPh-Dz concentration in a 96-well plate following the procedure described in section 1.3.1. At the indicated time points (2, 5, 10, 15, 30 minutes) 30 µL aliquots were taken for NIR fluorescence measurement (see section 1.1).

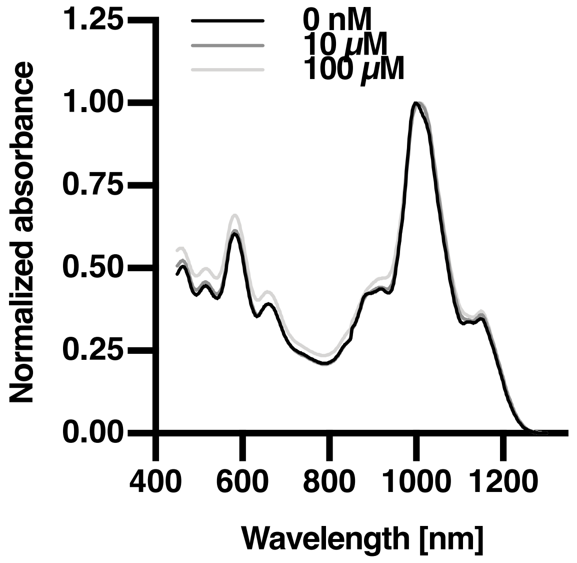

Figure S2. VIS/NIR absorbance spectroscopy of SDBS-dispersed SWCNTs before and after introduction of MalPh quantum defects [0, 10 and 100 µM MalPh-Dz **2** concentration].

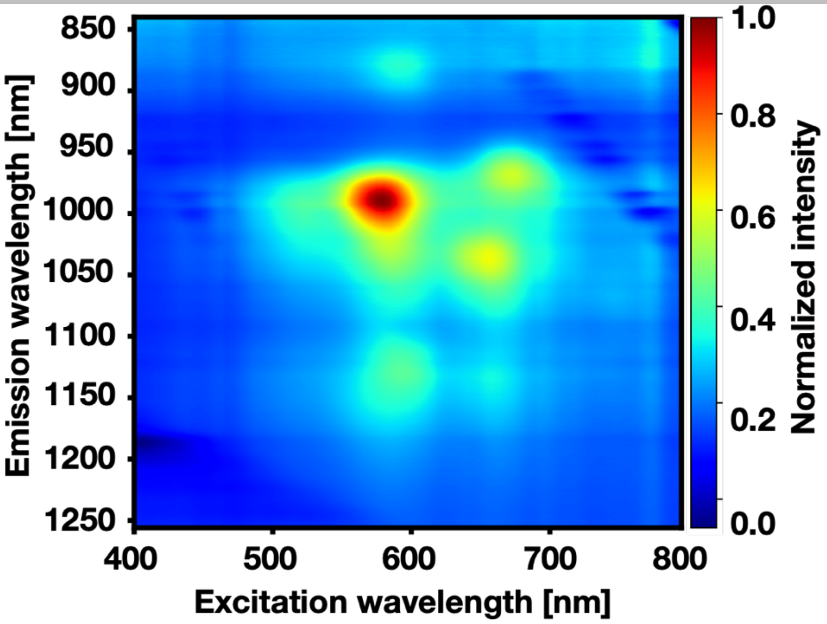

Figure S3. Excitation-emission map (2D spectrum) of SDBS-dispersed SWCNTs before introduction of MalPh quantum defects showing a major emission peak at approx. 1000 nm.

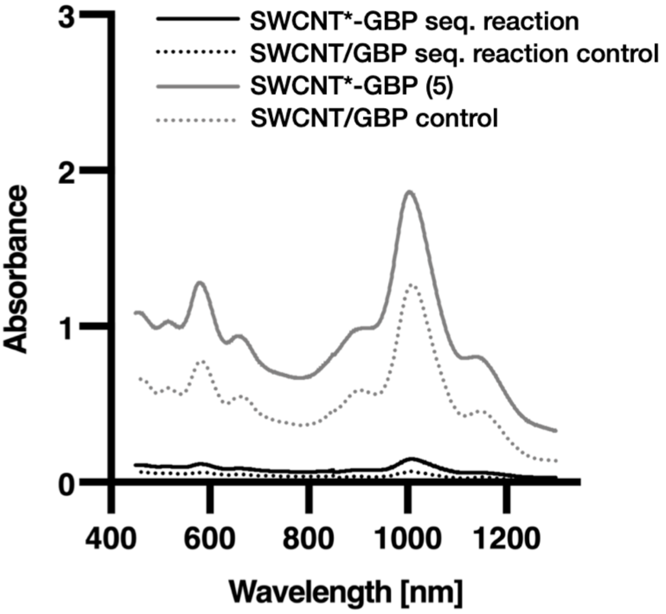

Figure S4. VIS/NIR absorbance spectroscopy of SWCNT*-GBP and the SWCNT/GBP control (comparing the sequential and the one-pot reaction) after removal of excess GBP using spin-filtration and redispersion in PL-PEG5000 of both positive and negative control of the GBP conjugation to SWCNTs. In contrast to the one-pot reaction (grey lines), redispersion of the samples originating from the sequential reactions did not lead to well-dispersed samples. The samples were prepared according to the procedures described in section 1.3.2 (for both controls without the addition of the MalPh diazonium salt and consequently without sp^3 defects).

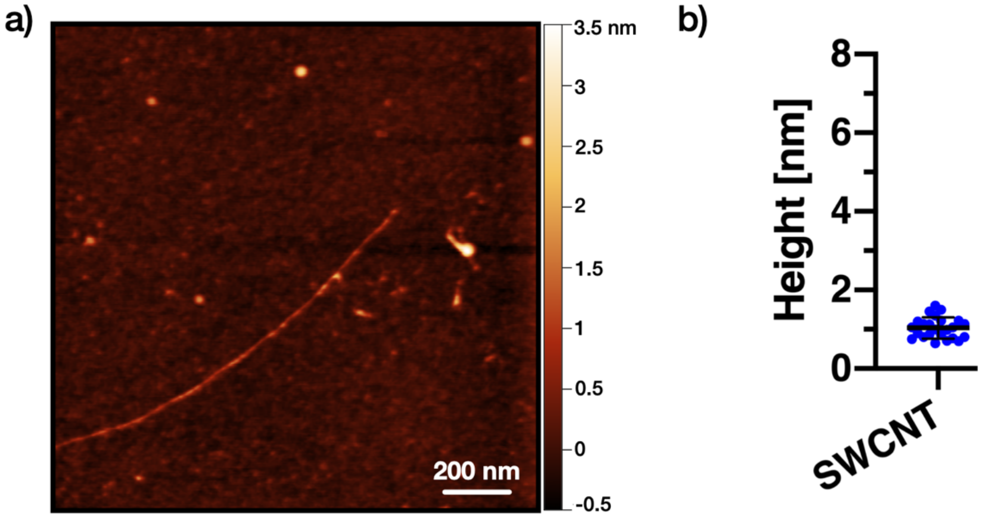

Figure S5. A) Representative atomic force microscopy image of a control SWCNT after following the same reaction steps as for conjugate **5** (one-step reaction, details see section 1.3.2.2) yet without the addition of MalPh-Dz **2** showing no conjugation of the nanobody. B) Measured heights of SWCNTs from the same control sample (mean ± SD, n = 24).

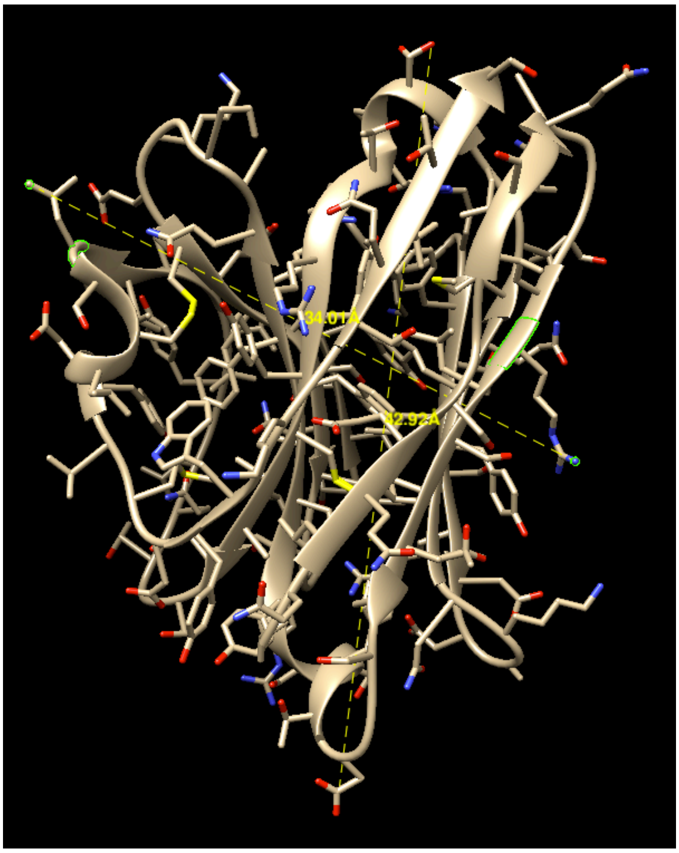

Figure S6. X-ray structure of a GFP-Minimizer nanobody (PDB: 3G9A) and distance measurements.

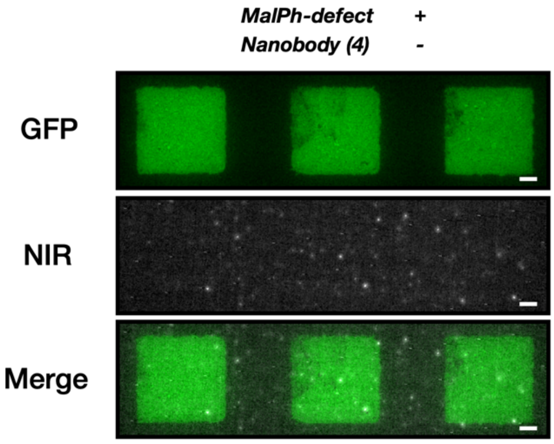

Figure S7. Control VIS/NIR fluorescence experiment with a microcontact-printed GFP-pattern incubated with MalPh-SWCNT* following the same procedure as described in section 1.4. The absence of colocalization in this experiment shows the importance of the nanobody for GFP-binding and rules out binding as a result from possibly unoccupied und unhydrolyzed MalPh groups on the SWCNT surface, that could be captured by GFP's thiols. Scale bars = 5 µm. The three columns are technical replicates.

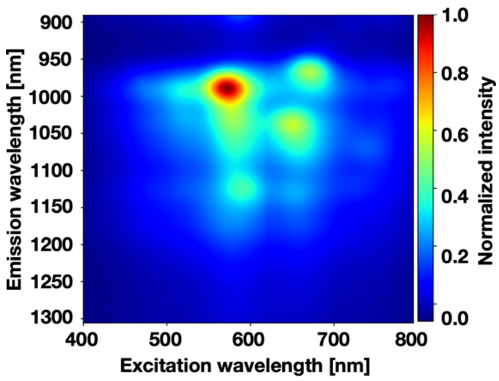

Figure S8. Excitation-emission map (2D spectrum) of a SWCNT/SDBS sample illuminated with green light for 15 minutes following the same procedure as for the synthesis of **7** (see section 1.3.3), but without the addition of Fmoc-Phe diazonium salt **6**.

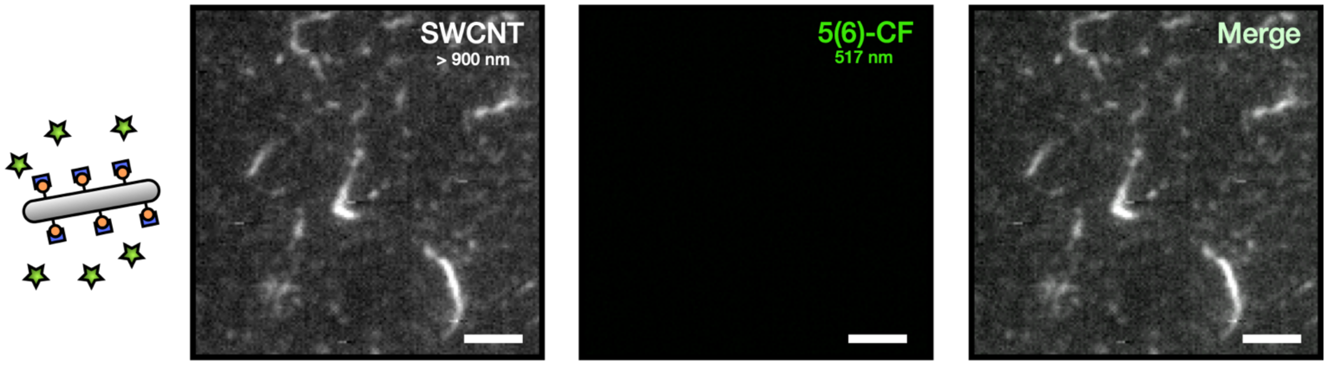

Figure S9. VIS/NIR fluorescence microscopy control experiment showing SWCNT*-F-Fmoc, that were treated using the same procedure as for the synthesis of **10** (see section 1.3.4) including the 5(6)-carboxyfluorescein coupling reaction, but without the Fmoc deprotection using 20% piperidine/DMF. After washing (see 1.3.4), they were immobilized on a glass surface and imaged. While they still show NIR fluorescence, no 5(6)-CF fluorescence can be observed and consequently no colocalization. Scale bars = 5 µm.

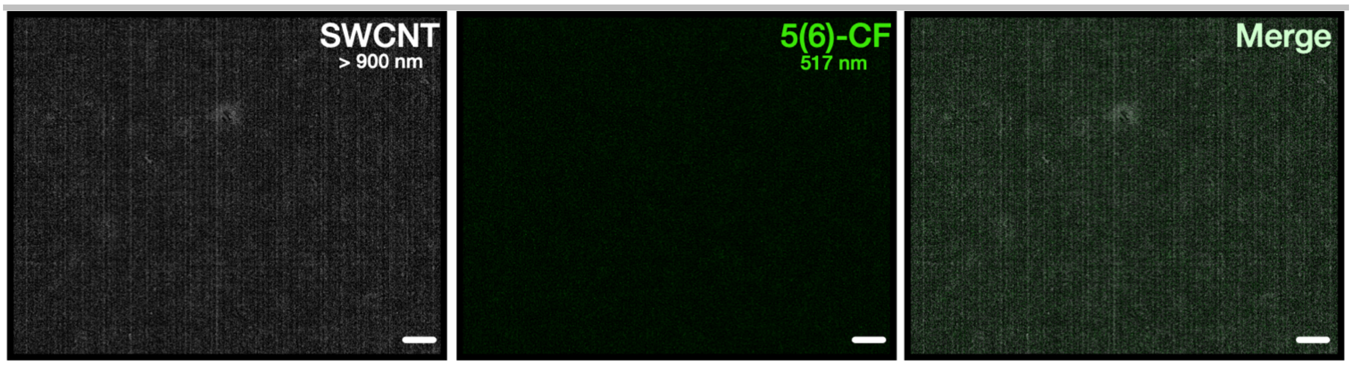

Figure S10. VIS/NIR fluorescence microscopy control experiment showing the SWCNT-R$_6$-CF control (without the addition of diazonium salt **6** and thus without F-Fmoc defects) after centrifugation and spin-coating on a glass coverslip showing neither carbon nanotube NIR-fluorescence nor CF fluorescence (scale bars = 10 µm).

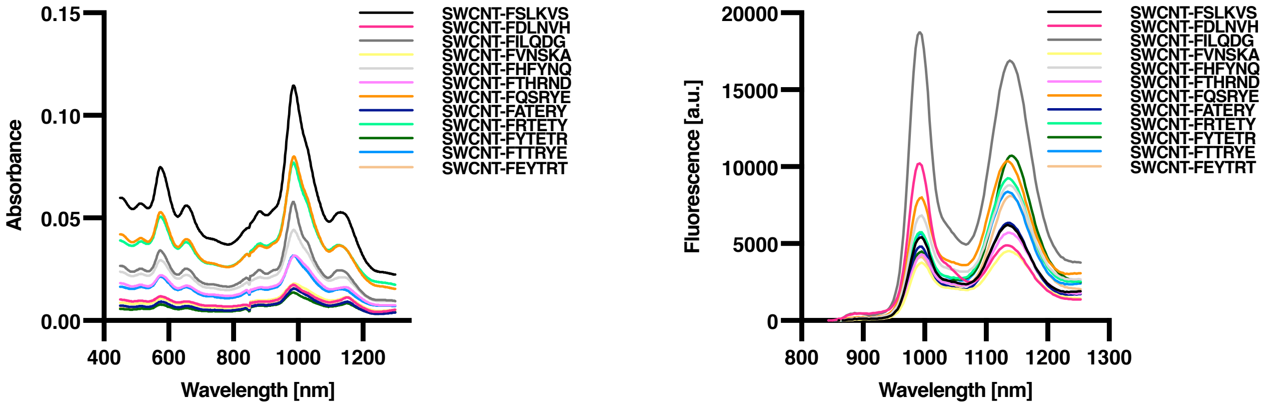

Figure S11. VIS/NIR absorbance and NIR fluorescence spectra of the SWCNT*-Peptides after redispersion using sodium deoxycholate (DOC). The absorbance intensity and thus also the absolute SWCNT concentrations are subject to variation due to small deviations in the tip-sonication process required for redispersion. For fluorescence measurements, the solutions were brought to the same SWCNT concentrations based on their absorbance at approx. 1000 nm (see section 1.1 for concentration determination).

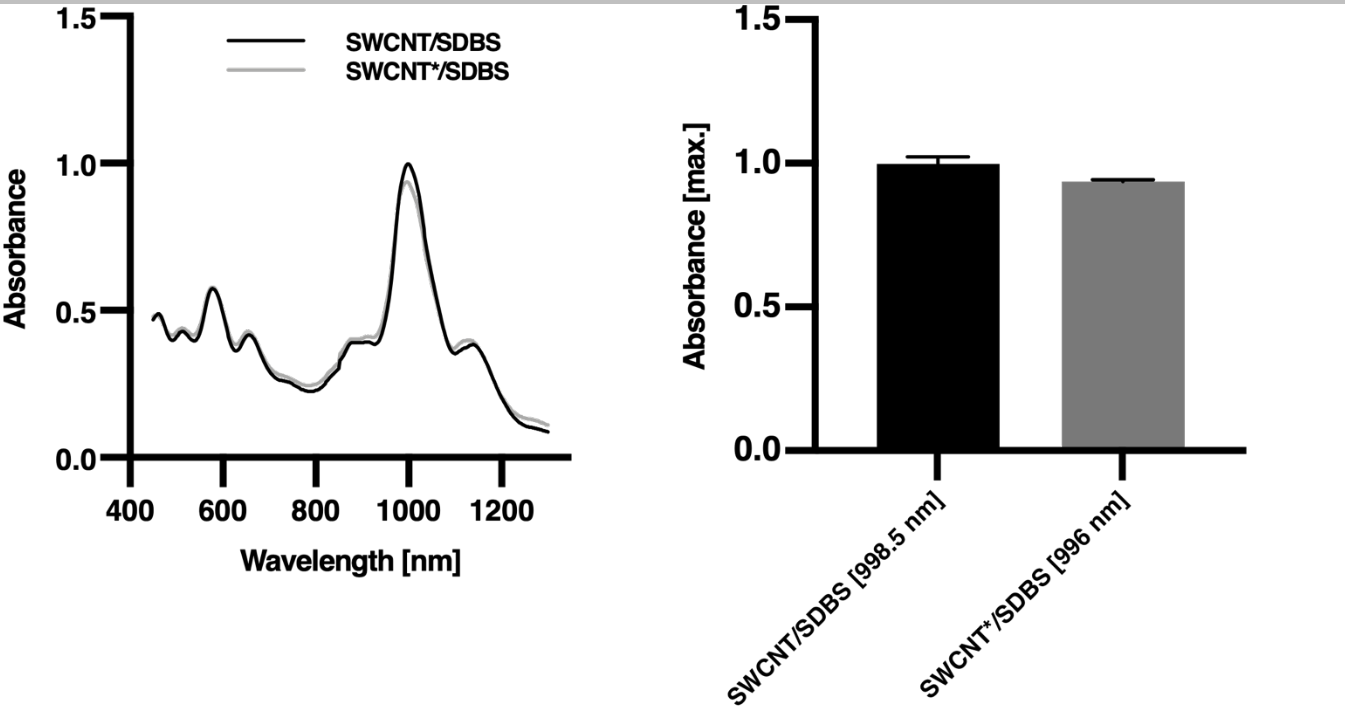

Figure S11. Absorbance spectra of a SWCNT/SDBS sample treated using green light for 15 minutes with (grey) and without the addition of diazonium salt **6** (black) measured in the supernatant after centrifugation (30 min, 16100g). Mean ± SD (*N* = 3).

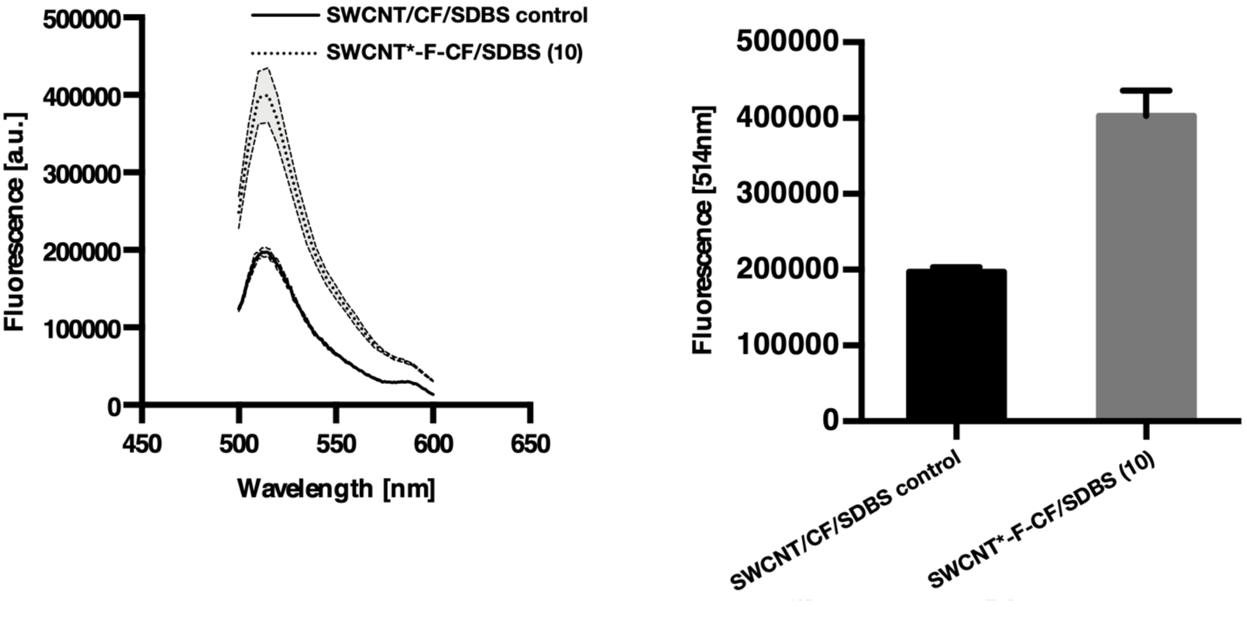

Figure S12. Fluorescence spectroscopy of positive and negative control samples for the conjugation of 5(6)-CF to SWCNT*-F-NH_2. The control was generated following the same procedure as for the synthesis of **10** (see section 1.3.4), yet without prior F-Fmoc defect introduction. Despite some degree of background fluorescence also in the control sample, which might arise from non-covalent adsorption to the hydrophobic SWCNT surface, the positive sample having the F-defect shows a significantly higher CF-fluorescence. Mean ± SD (*N* = 3).

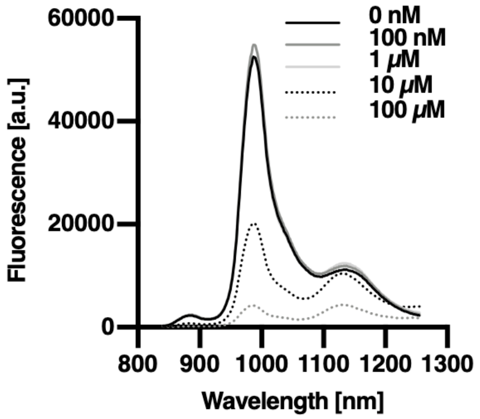

Figure S13. Absolute NIR fluorescence spectra of SWCNTs before and after introduction of MalPh defects with different concentrations of MalPh-Dz (15 minutes illumination time) showing the decrease of the E_{11} with increasing MalPh-Dz concentration.

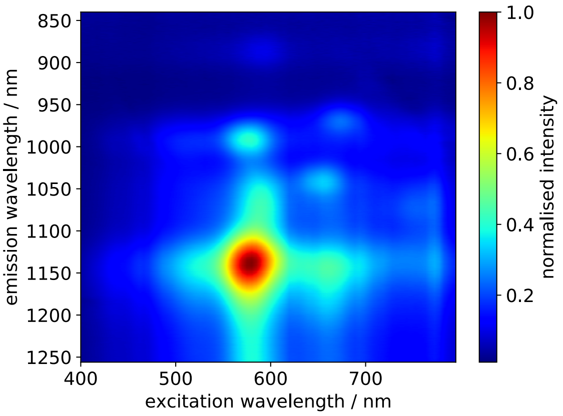

Figure S14. Excitation-emission map (2D spectrum) of a MalPh-SWCNT* sample after spin-filtration and redispersion using PL-PEG5000 following the process described in section 1.3.1.

3. References

[1] F. Schöppler, C. Mann, T.-C. Hain, F.-M. Neubauer, G. Privitera, F. Bonaccorso, D. Chu, A.-C. Ferrari, T. Hertel, *J. Phys. Chem. C* **2011**, 115, 14682-14686.

[2] K. Zhang, X.-H. Xu, F.L. Qing, *Eur. J. Org. Chem.* **2016**, *30*, 5088–5090.

[3] J.-C. Harper, R. Polsky, D.-R. Wheeler, S.-M. Brozik, *Langmuir* **2008**, *24*, 2206-2211.

4. Author Contributions

S.K. and F.A.M. designed and conceived the project. N.H. performed synthesis of the MalPh diazonium salt, NMR/MS analysis, initial experiments and optimized the conditions for MalPh-defect incorporation. F.A.M. performed AFM experiments, synthesized the Fmoc-Phe diazonium salt with N.H., optimized the conditions for Fmoc-Phe defect incorporation, performed the nanobody conjugation and validation experiments, synthesized multi-color SWCNTs, SWCNT-R_6 and performed the 96-well SWCNT-Peptide synthesis as well as the subsequent characterization. F.O. provided the GFP-binding nanobody. F.A.M., N.H. and S.K wrote the manuscript with input from F.O.

5. NMR spectra of diazonium salts 2 and 6

5.1. 4-(*N*-maleimido)phenyldiazonium tetrafluoroborate (2)

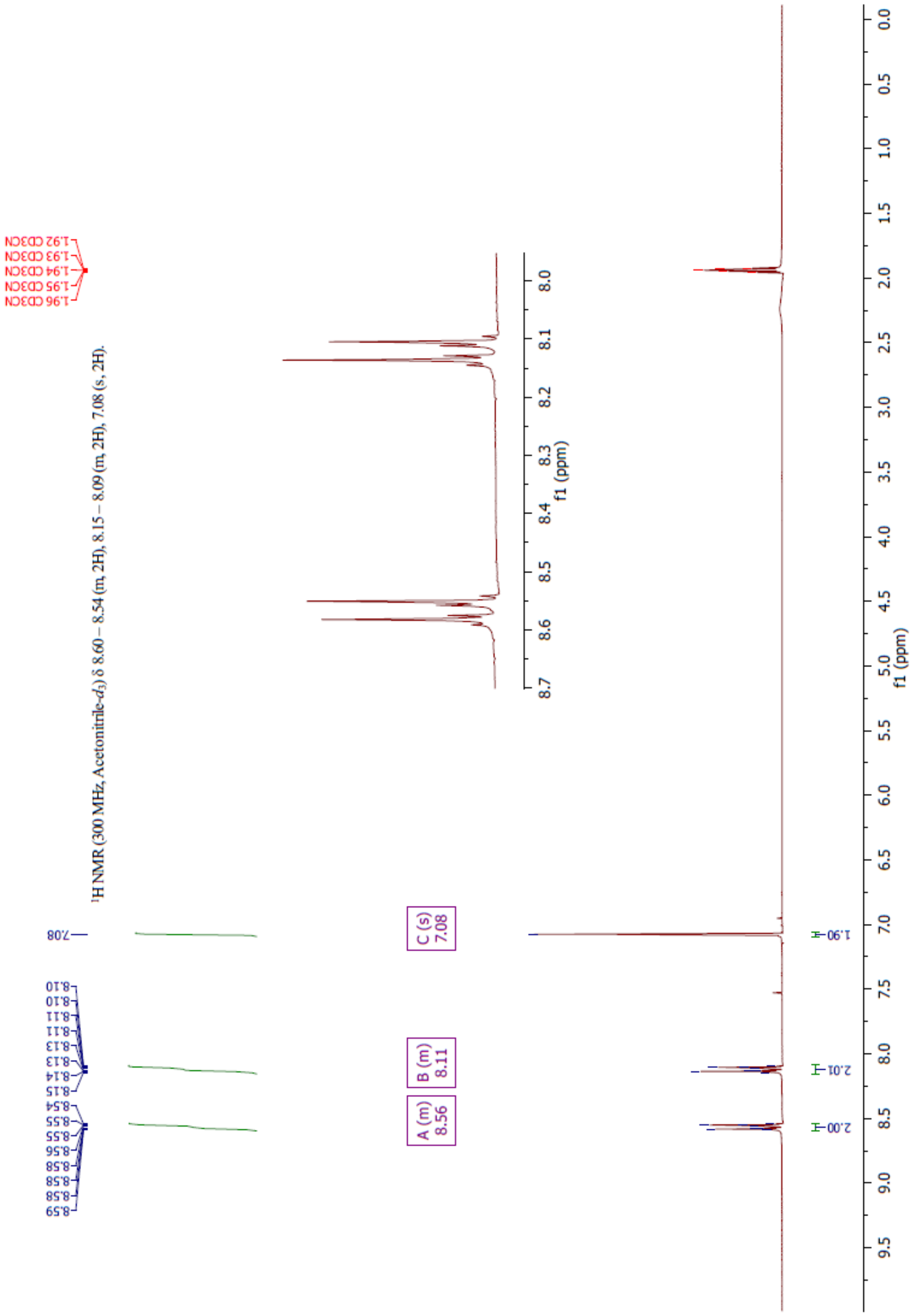

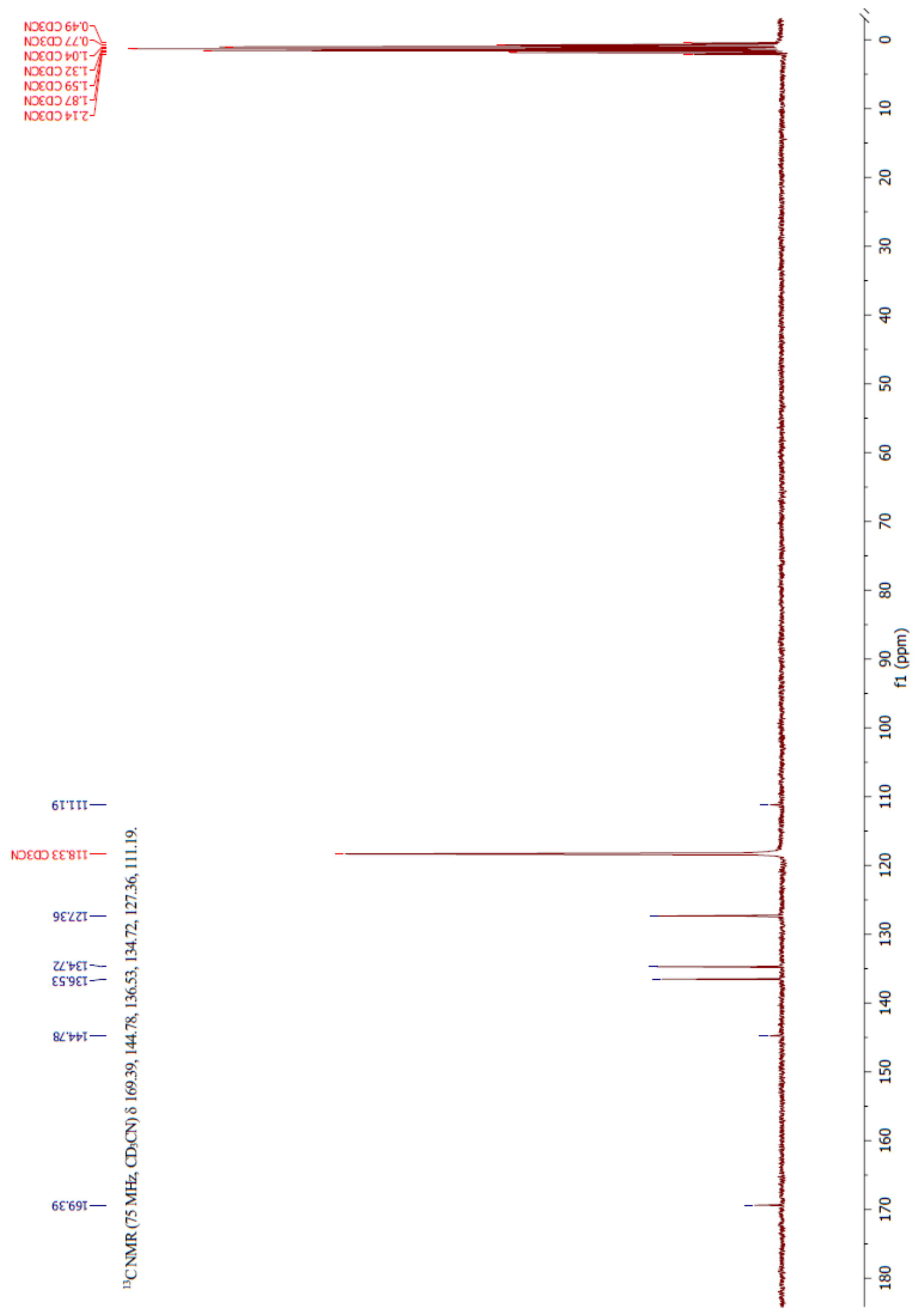

^{13}CNMR (75 MHz, CD_3CN) δ 169.39, 144.78, 136.53, 134.72, 127.36, 111.19.

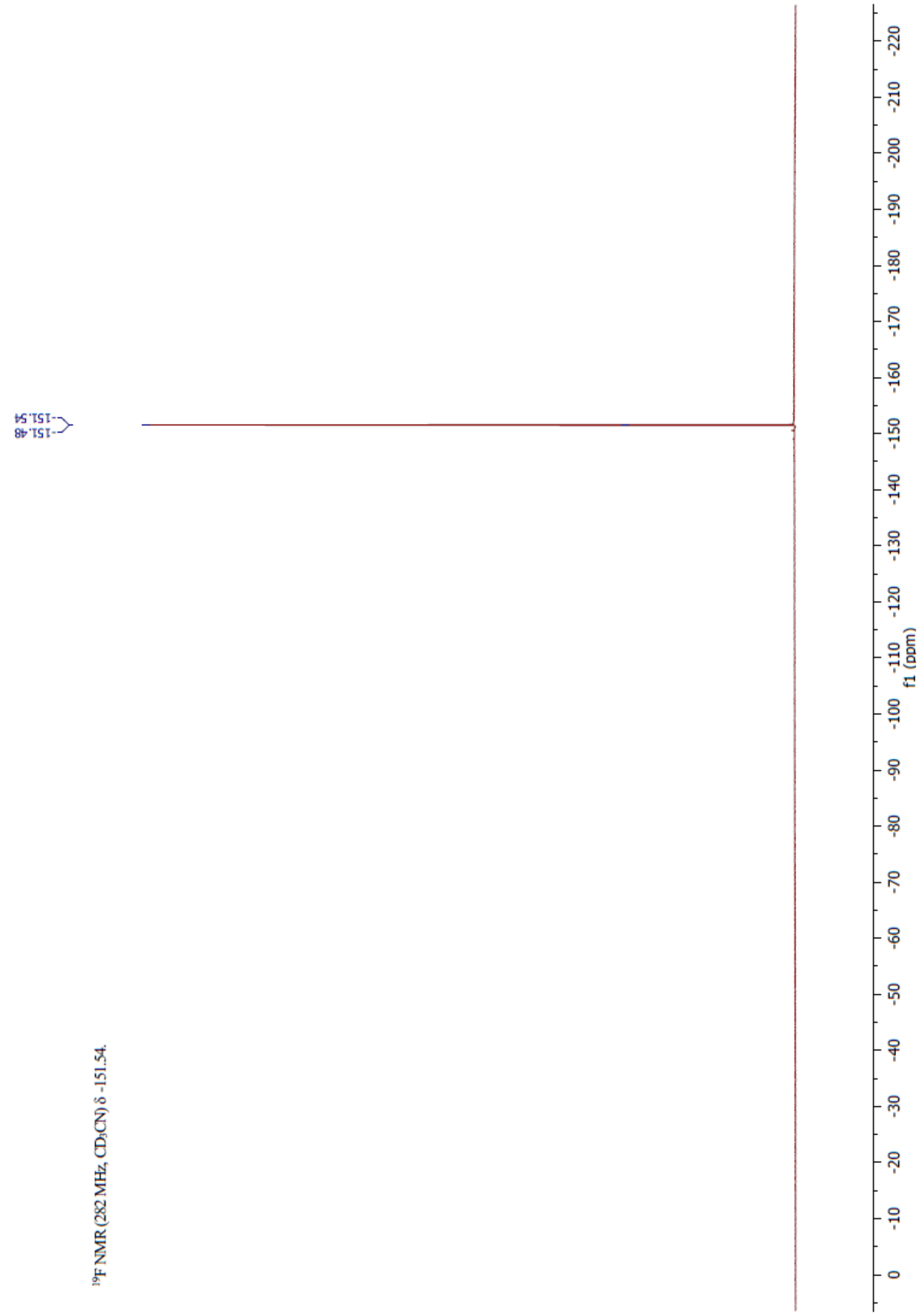

^{19}F NMR (282 MHz, CD_3CN) δ -151.54.

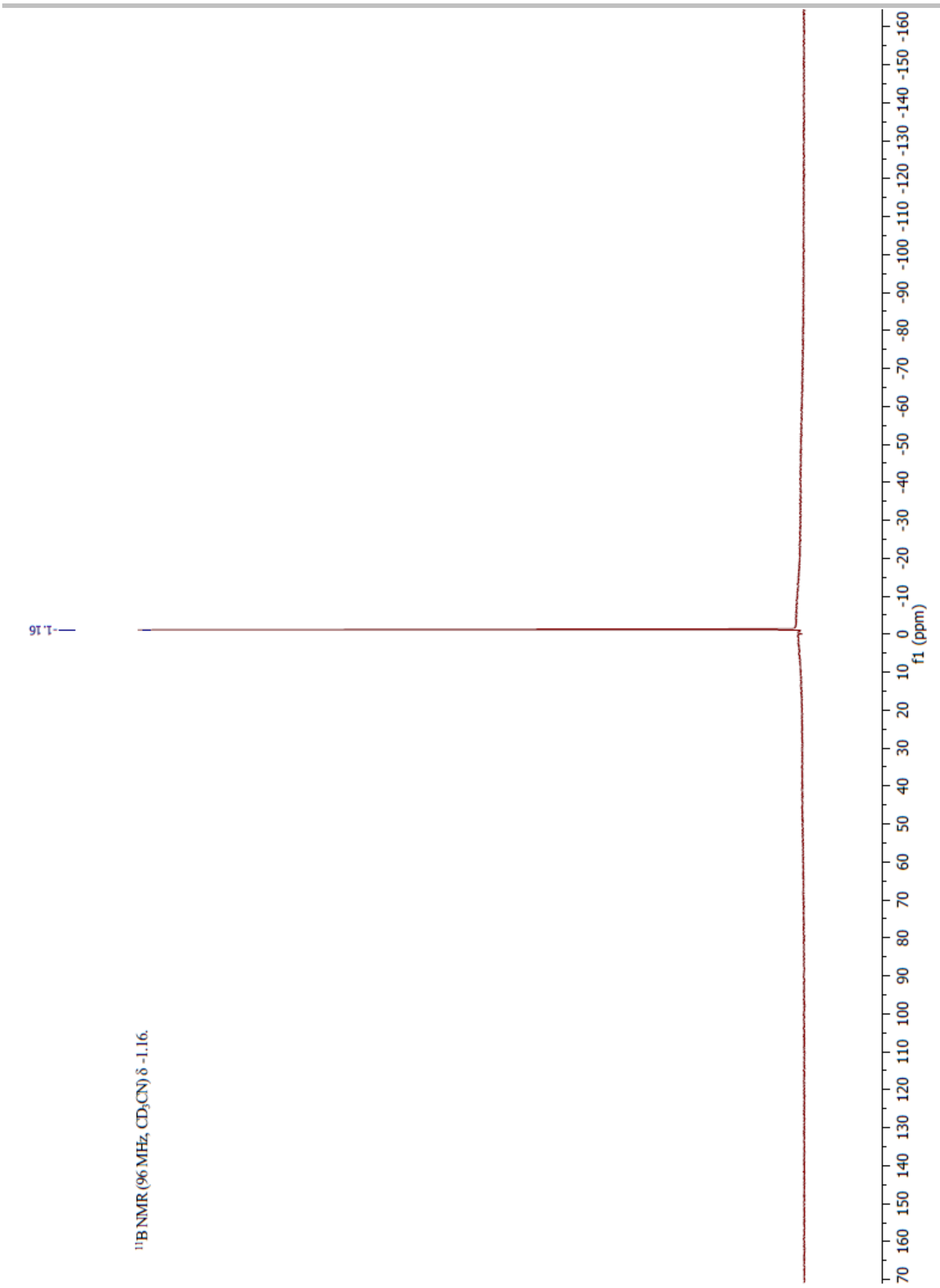

^{11}B NMR (96 MHz, CD_3CN) δ -1.16.

5.2. Fmoc-L-4-diazonium-phenylalanine tetrafluoroborate (6)

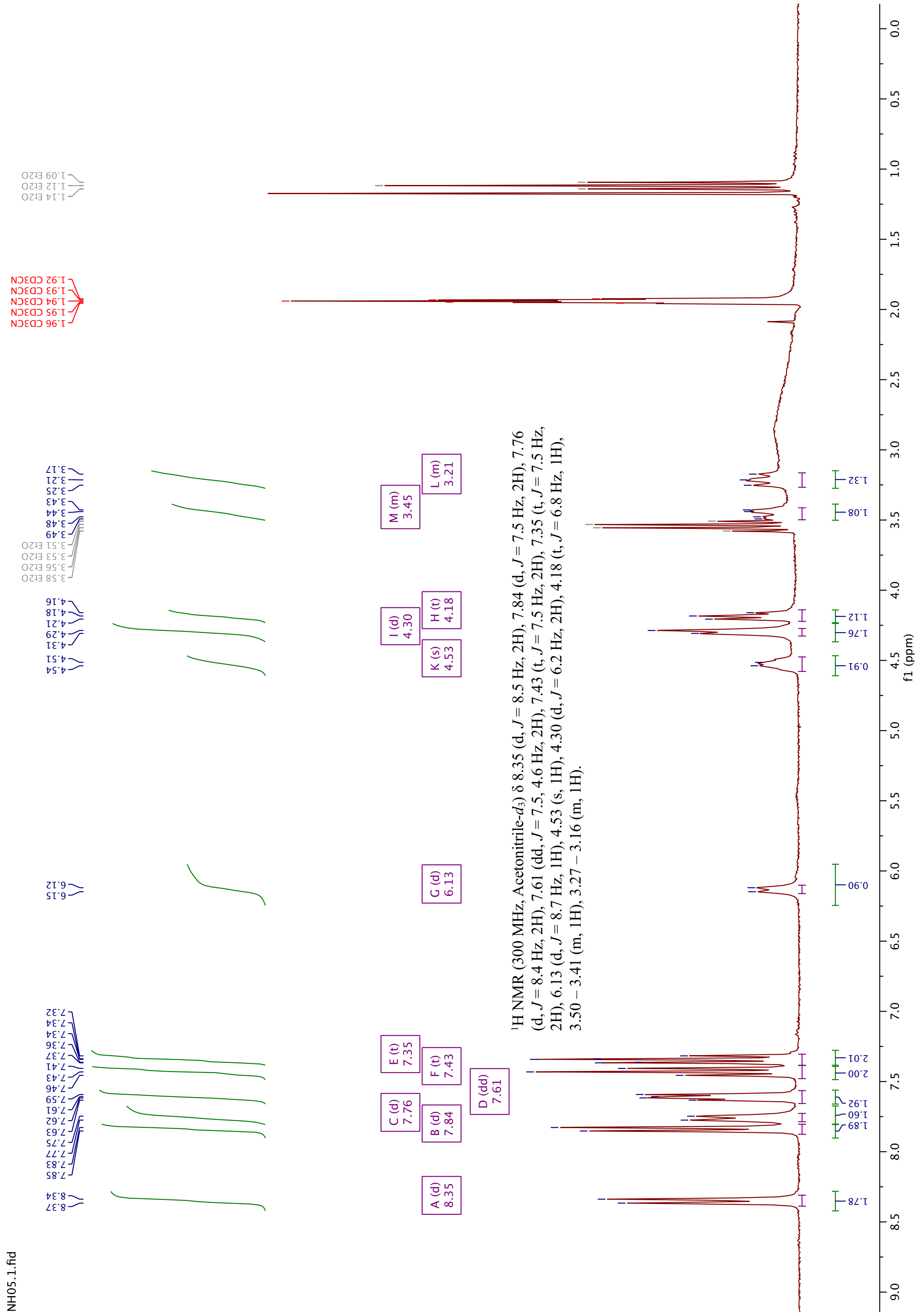

^{1}H NMR (300 MHz, Acetonitrile-d_3) δ 8.35 (d, J = 8.5 Hz, 2H), 7.84 (d, J = 7.5 Hz, 2H), 7.76 (d, J = 8.4 Hz, 2H), 7.61 (dd, J = 7.5, 4.6 Hz, 2H), 7.43 (t, J = 7.5 Hz, 2H), 7.35 (t, J = 7.5 Hz, 2H), 6.13 (d, J = 8.7 Hz, 1H), 4.53 (s, 1H), 4.30 (d, J = 6.2 Hz, 2H), 4.18 (t, J = 6.8 Hz, 1H), 3.50 – 3.41 (m, 1H), 3.27 – 3.16 (m, 1H).

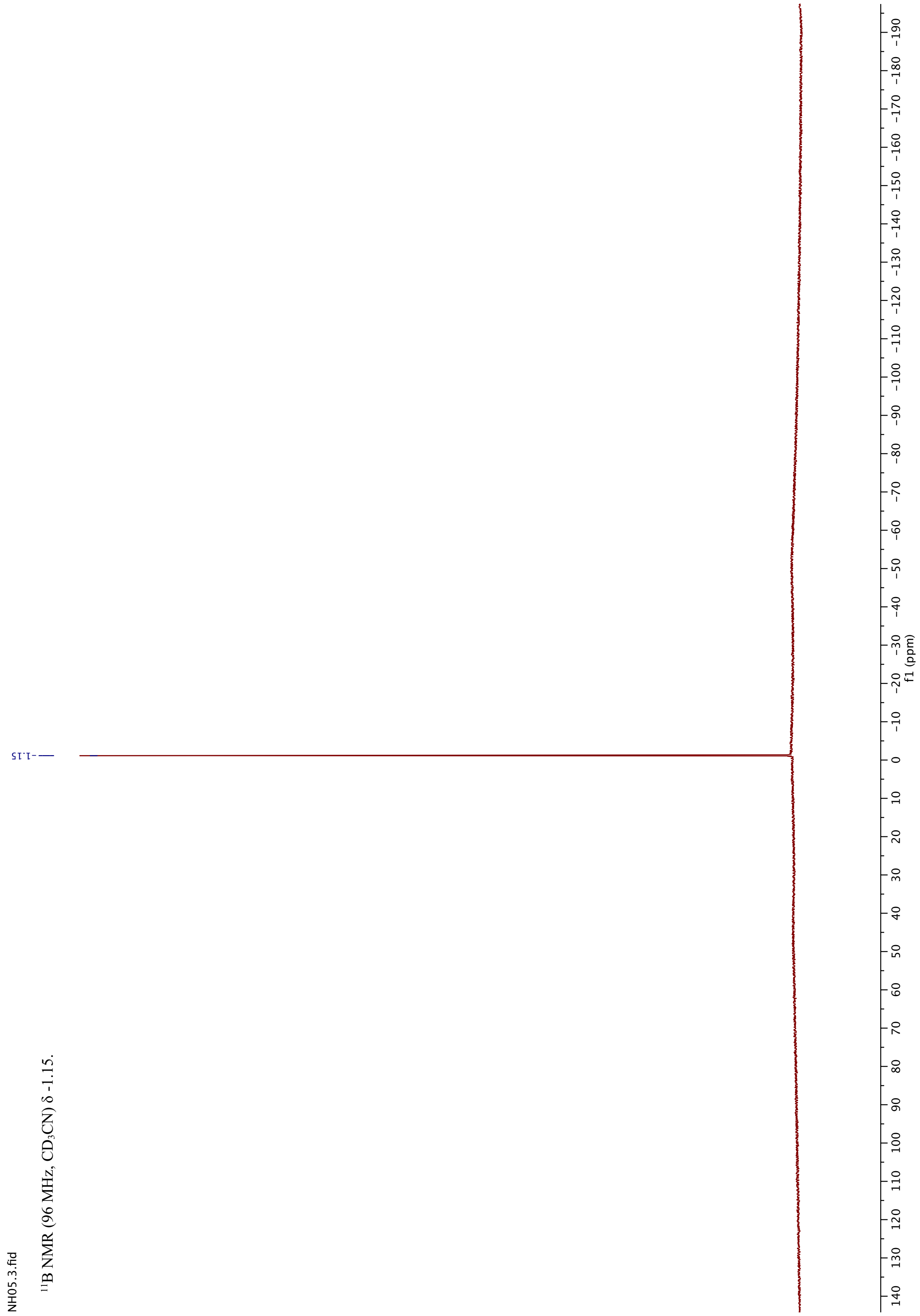

^{11}B NMR (96 MHz, CD_3CN) δ -1.15.

NH05.2.fid

^{19}F NMR (282 MHz, CD_3CN) δ -151.47, -151.53.

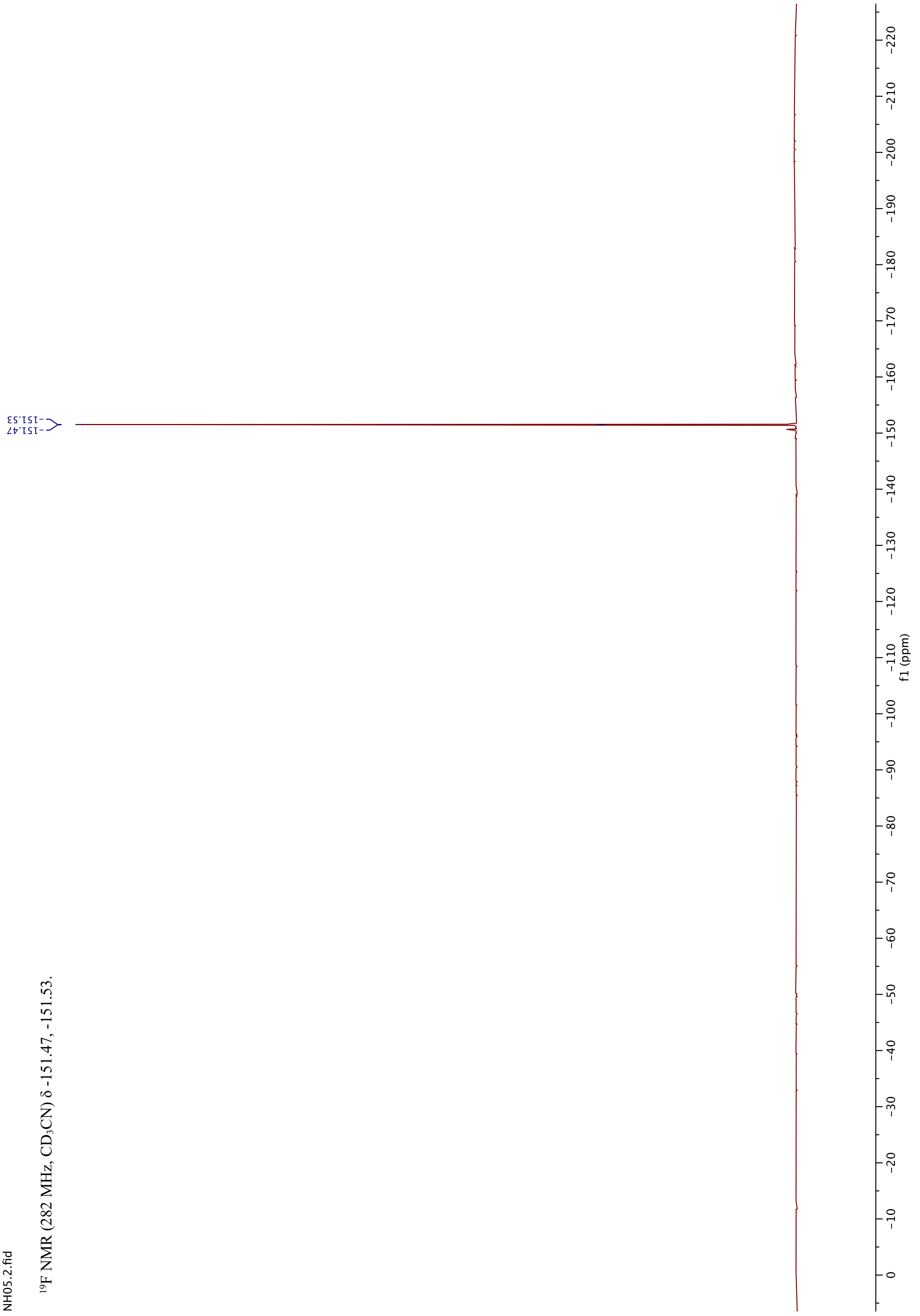

Supplement: Supplementary file 1 — Supplementary [file ANIE-59-17732-s001.pdf]
